# MHC-related protein 1–restricted recognition of cancer via a semi-invariant TCR-**α** chain

**DOI:** 10.1172/JCI181895

**Published:** 2025-01-02

**Authors:** Garry Dolton, Hannah Thomas, Li Rong Tan, Cristina Rius Rafael, Stephanie Doetsch, Giulia-Andreea Ionescu, Lucia F. Cardo, Michael D. Crowther, Enas Behiry, Théo Morin, Marine E. Caillaud, Devinder Srai, Lucia Parolini, Md Samiul Hasan, Anna Fuller, Katie Topley, Aaron Wall, Jade R. Hopkins, Nader Omidvar, Caroline Alvares, Joanna Zabkiewicz, John Frater, Barbara Szomolay, Andrew K. Sewell

**Affiliations:** 1 Division of Infection and Immunity, Cardiff University School of Medicine, Cardiff, United Kingdom.; 2 Nuffield Department of Medicine and Department of Chemistry, University of Oxford, Oxford, United Kingdom.; 3 Division of Cancer and Genetics, Cardiff University School of Medicine, Cardiff, United Kingdom.; 4 Nuffield Department of Medicine and NIHR Biomedical Research Centre University of Oxford, Oxford, United Kingdom.; 5 Systems Immunology Research Institute, Cardiff University Cardiff, United Kingdom.; 6 Division of Infection and Immunity, Kumamoto University, Kumamoto, Japan.

**Keywords:** Immunology, Oncology, Cancer, Cellular immune response, T cells

## Abstract

The T cell antigen presentation platform MR1 consists of 6 allomorphs in humans that differ by no more than 5 amino acids. The principal function of this highly conserved molecule involves presenting microbial metabolites to the abundant mucosal-associated invariant T (MAIT) cell subset. Recent developments suggest that the role of MR1 extends to presenting antigens from cancer cells, a function dependent on the K43 residue in the MR1 antigen binding cleft. Here, we successfully cultured cancer-activated, MR1-restricted T cells from multiple donors and confirmed that they recognized a wide range of cancer types expressing the most common MR1*01 and/or MR1*02 allomorphs (over 95% of the population), while remaining inert to healthy cells including healthy B cells and monocytes. Curiously, in all but one donor these T cells were found to incorporate a conserved TCR-α chain motif, CAXYGGSQGNLIF (where X represents 3–5 amino acids), because of pairing between 10 different *TRAV* genes and the *TRAJ42* gene segment. This semi-invariance in the TCR-α chain is reminiscent of MAIT cells and suggests recognition of a conserved antigen bound to K43.

## Abstract 2

The first description of a new subtype of T-cell that occur in most individuals and that can kill most types of cancer.

## Graphical abstract

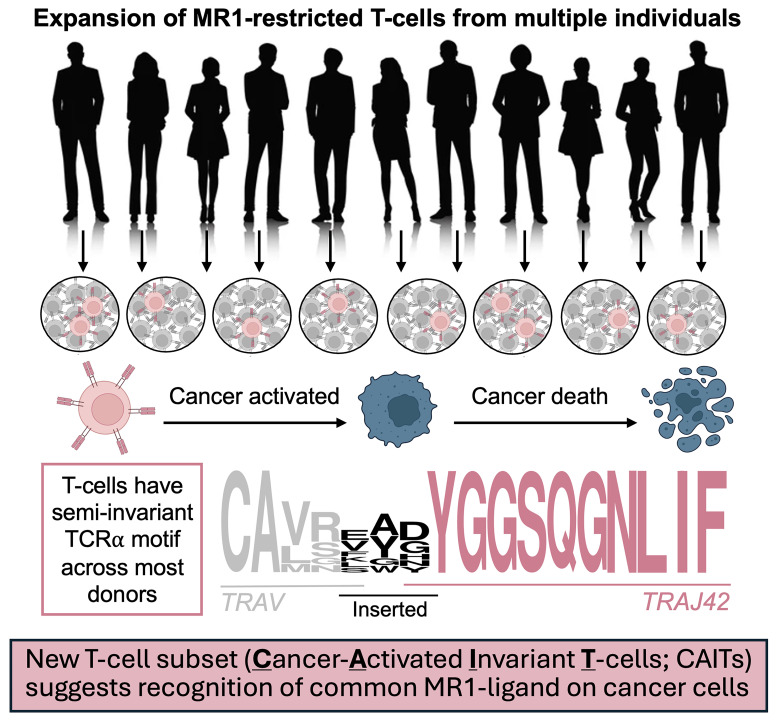

## Introduction

Cytotoxic T lymphocytes (CTL), often called killer T cells, can destroy cells harboring intracellular pathogens or dysregulated gene expression associated with cancerous transformation. Classically, CTL express the CD8 glycoprotein and recognize processed proteinaceous antigens as short peptides of 8–14 amino acids in length presented by major histocompatibility complex class I (MHC-I) molecules (1). CD8 engages a largely invariant site on MHC-I to augment recognition of the peptide MHC by the highly variable αβ T cell receptor (TCR) (2, 3). More recent work has shown that some αβ T cells can recognize nonprotein, foreign, intracellular antigens presented by the CD1 family of proteins and MHC-related protein 1 (MR1), which also assemble with β2-microglobulin and adopt a highly similar fold and structure to MHC-I (4, 5). In humans CD1a, CD1b, CD1c, and CD1d are known to present different lipid antigens to T cells, whereas MR1 presents microbial metabolites to mucosal-associated invariant T (MAIT) cells (5, 6). MR1 has been conserved throughout mammalian evolution to an extent where human MAIT cells can cross react with murine MR1-presenting antigen (7, 8). MR1 is known to present vitamin B metabolites to MAIT cells and thereby signal that a self cell harbors an internal microbe (9, 10). The MAIT cell subset is characterized by a TCR-α chain that is made up of the *TRAV1-2* gene in combination with a *TRAJ33*, *TRAJ12*, or *TRAJ20*-joining region (11). This semi-invariant TCR-α chain combines with a restricted number of TCR-β chains to severely limit MAIT cell TCR variability compared with αβ TCRs that recognize MHC-I (12, 13). Unlike human MHC-I, where there are over 10,000 different human leukocyte antigen class I (HLA-I) alleles across the human population (14), MR1 is far less variable with minimal amino acid differences, most of which are distal to the TCR docking site (15). A study of 56 samples with diverse HLA genotypes found 6 allele groups encoding for different but highly similar MR1 proteins. The vast majority of the study sample expressed either MR1*01 (71%) or MR1*02 (25%), which differ by encoding a histidine and an arginine at position 17, respectively, a position not thought to affect either antigen or TCR binding (15). Less than 3 amino acid substitutions were associated with the infrequent MR1*03 (I121V), MR1*04 (R9H, H17R), MR1*05 (E52G, H90Q, I244V), and MR1*06 (R304K) alleles. Of these substitutions, only R9H, present in MR1*04, is close to the known MR1 antigen binding site. Interestingly, a recent report described an individual who was homozygous for the R9H variant of MR1 that had a primary immunodeficiency and was found not to possess the MAIT cell subset (16). The R9H variant protein failed to present 5-(2-oxopropylideneamino)-6-d-ribitylaminouracil (5-OP-RU) to MAIT cells, providing a possible explanation for the lack of MAIT cells in this individual. It is not yet known whether other allelic variants of MR1 affect protein function or expression levels. Recent studies have shown that MR1 can present self antigens to MR1-restricted T cells (17) with a single MR1-restricted T cell being able to kill a wide range of cancer cell lines and primary cancer cells while remaining inert to healthy cells (18). The limited allelic variation of MR1 compared with HLA makes it an attractive target for potential cancer therapies. Here, we searched for cancer-activated, MR1-restricted T cell populations in 10 healthy donors and a patient with acute myeloid leukemia (AML). All donors possessed T cell populations that activated in response to MR1^+^ cancer cell lines but not MR1-KO lines. These T cells expressed CD8 and showed some dependency on lysine 43, which is known to form a Schiff base with bacterial MR1 ligands (19). TCR sequencing revealed a conserved TCR-α chain CAXYGGSQGNLIF (where X represents 3–5 amino acids) motif in 10 of 11 individuals that used the *TRAJ42* gene that included the *TRAJ42*-encoded tyrosine residue at position 94–99, depending on *TRAV* gene usage. A similar conservation of TCR-α sequence, including a conserved Y95-α residue, is seen in MAIT cells (20), and suggests that *TRAJ42*^+^ cancer-activated invariant T cells are likely to see a common ligand.

## Results

### Generation of cancer-reactive MR1-restricted T cell lines and clones from multiple donors.

**Figure 1:**
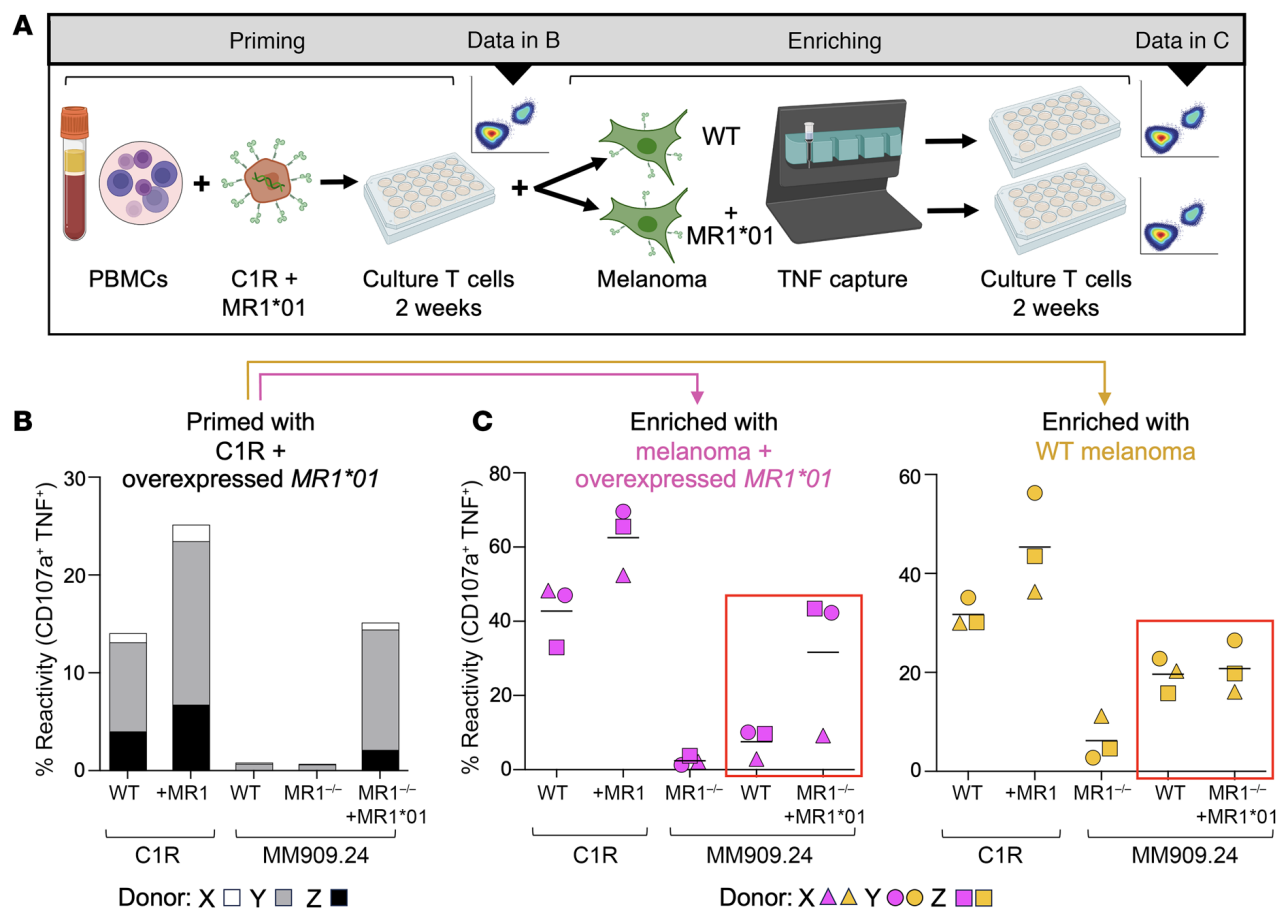
Induction of T cells reactive to natural levels of MR1 on the surface of cancer cells. (**A**) PBMCs from healthy donors were primed for 2 weeks with C1R cells overexpressing MR1*01. Primed T cells were enriched for reactivity toward WT melanoma,or MR-KO (^–/–^) melanoma cells overexpressing MR1*01, using TNF capture antibody and magnetic beads. The captured T cells were expanded for 2 weeks with irradiated PBMCs and PHA before analysis. (**B**) Three donors primed for 2 weeks with C1R cells overexpressing MR1*01 then tested against C1R and melanoma MM909.24 cells, indicated on the x-axis. Intracellular cytokine staining (ICS) was performed with CD107a and TNF antibodies. (**C**) The primed lines from B were coincubated with WT melanoma MM909.24 or MR1-KO melanoma MM909.24 overexpressing MR1*01, then reactive T cells captured based on TNF secretion and magnetic sorting. T cells enriched with the melanoma overexpressing MR1*01 (pink) preferred the overexpressed cell line (red box), whereas the T cells enriched with WT melanoma (yellow) gave similar MR1-dependent reactivity for both the WT and MR1 overexpressing melanoma (red box). ICS was performed with CD107a and TNF antibodies.

We recently discovered an MR1-restricted T cell clone with an αβ TCR that allowed it to target a wide range of cancer cell lines and also control human leukemia cells in an NSG mouse model (18). Importantly, transfer of the TCR from this clone to the autologous CD8^+^ T cells from 2 patients with melanoma allowed these cells to lyse the patient’s own tumor line (18). This finding has garnered interest in exploring MR1-mediated options for immunotherapy due to the substantially lower variability of MR1 across the population compared with HLA (21, 22) (Supplemental Figure 1; supplemental material available online with this article; https://doi.org/10.1172/JCI181895DS1). We set out to generate further T cell clones that responded to cancer lines via MR1 to determine whether most donors had T cells with this property. Our catch-all strategy to generate cancer-specific MR1-restricted T cells is outlined in Figure 1A and involved T cell priming from the PBMC of 3 donors using C1R cells (MR1*01^+/+^) overexpressing MR1*01. After the initial priming, 2 of 3 donors had T cells reactive toward overexpressed MR1 on either C1R or melanoma cells, but no MR1-restricted reactivity with WT melanoma cells (Figure 1B). These primed T cells were then enriched following exposure to WT melanoma line MM909.24 or MR1-KO MM909.24 overexpressing MR1*01 and expanded for 2 weeks prior to testing. Two of the lines enriched with MM909.24 overexpressing MR1*01 responded well to these targets but exhibited dependency on overexpressed MR1*01 for maximal reactivity, with minimal activation toward WT melanoma cells (Figure 1C). In contrast, the lines from all 3 donors that were enriched with WT MM909.24 displayed similar reactivity to WT MM909.24 and MR1*01 overexpressed cells, while not responding to MM909.24 *MR1^–/–^* targets (Figure 1C). These experiments show that it is possible to culture T cells that recognize a WT melanoma cell line via the MR1 molecule from all donors tested. We next aimed to characterize these T cells and the TCRs they express.

### Characterization of MR1-restricted melanoma reactive T cells.

**Figure 2:**
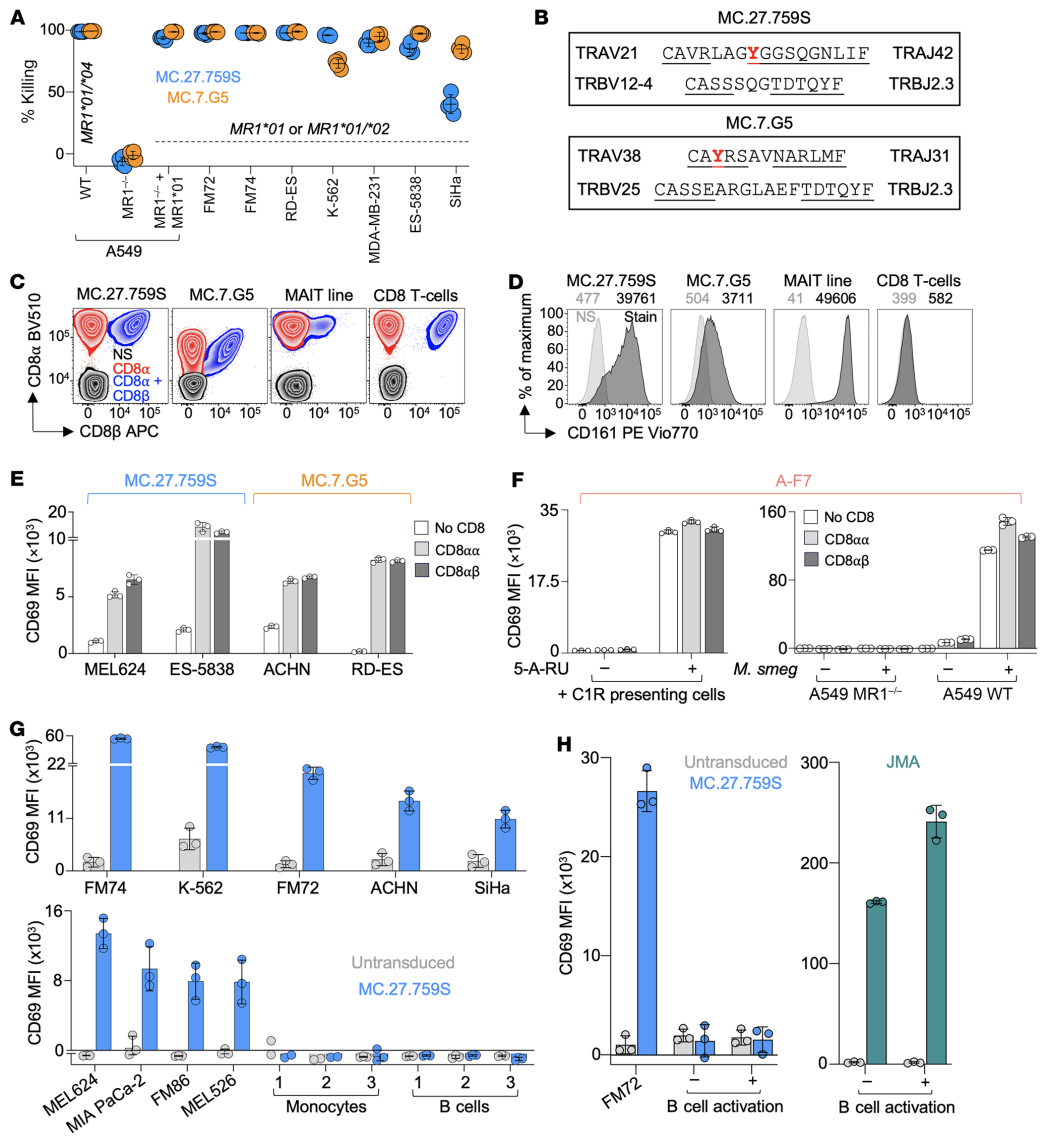
Characterization of the MC.27.759S T cell receptor. (**A**) MC.27.759S and MC.7.G5 T cell clone killing assay of various cancer cells (MR1 allotype indicated). A549 (lung) MR1 KO (^–/–^) by CRISPR/Cas9, with a transgene for scβ2M-MR1*01. Flow cytometry–based killing assay at a 1:2 T cell–to–cancer cell ratio for 24 hours. Quadruplicate conditions with error bars depicting SD. (**B**) TCR-α and -β chains of MC.27.759S and MC.7.G5. Germline amino acid residues of variable (V) and joining (J) regions are underlined and inserted amino acids not underlined. (**C**) CD8 phenotyping of MC.27.759S and MC.7.G5 T cell clones with CD8-α and CD8-β–specific antibodies. MAIT and CD8^+^ T cell line used as a controls for CD8-αα or CD8-αβ expression. (**D**) CD161 phenotyping of MC.27.759S and MC.7.G5 T cell clones, using MAIT and CD3/CD28-amplified T cells as in **C**. The MFI of CD161 staining is shown. (**E**) CD69 assays for 24 hours of MC.27.759S and MC.7.G5 TCR-transduced Jurkat cells expressing no CD8, CD8-αα or CD8-αβ, versus cancer cells. Untransduced values have been subtracted. Triplicate conditions with errors bars depicting SD. (**F**) CD69 assays for 24 hours of A-F7 (MAIT) TCR-transduced Jurkat cells expressing no CD8, CD8-αα or CD8-αβ versus *M*. *smeg–*infected A549 cells or 100 μg of 5-A-RU. Untransduced values have been subtracted. Triplicate conditions with errors bars depicting SD. (**G**) CD69 assays (24 hours) of MC.27.759S TCR-transduced Jurkat cells against MR1*01/*01 or *01/*02 cancer cells and healthy cells. Duplicate for monocytes from donors 1 and 2 due to the low number of cells available, or triplicate conditions with errors bars showing SD. (**H**) CD69 assay (24 hours) with MC.27.759S or JMA (positive control for MR1 on healthy cells) TCR-transduced Jurkat cells versus melanoma and inactivated or activated B cells. Triplicate conditions with errors bars showing SD. Cancer cells include melanoma (FM74, FM72, MEL624, MEL526, and FM86), Ewing sarcoma (RD-ES and ES-5838), Leukemia/CML (K-562), cervical (SiHa), breast (MDA-MB-231), kidney (ACHN) and pancreatic (MIA PaCa-2) cancers.

We succeeded in isolating an MR1-restricted T cell clone from Donor 0, called MC.27.759S, which grew sufficiently in culture to allow us to make comparisons with the previously described T cell clone MC.7.G5 (18). MC.27.759S and MC.7.G5 both killed A549 WT (MR1*01/*04) lung cancer cells but failed to kill these cells when MR1 was knocked out using CRISPR technology (23) (Figure 2A). Expression of MR1*01 as a single chain (sc) β2M-MR1 protein in the MR1-KO cancer cells restored killing by MC.27.759S (Figure 2A). We also tested 7 MR1*01/*01 or *01/*02 melanoma (FM72 and FM74), breast (MDA-MB-231), Ewing sarcoma (RD-ES and ES-5838), leukemia (K-562), and cervical (SiHa) cancer lines in the same killing assay (*MR1* genotypes of cancer cells in Supplemental Figure 2). Killing by MC.27.759S ranged between 96.1% and 98.9% for A549, FM72, FM74, RD-ES, and K-562 cell lines, and, for MC.7.G5, killing was between 95% and 98.4% for cell lines A549, FM72, FM74, ES-5838, RD-ES, and MDA-MB-231 (Figure 2A). There were some differences between the 2 clones, as MC.27.759S was better at killing K-562 than MC.7.G5 (96.1 % versus 72.9%), and MC.7.G5 seemed to prefer SiHa target cells (84.9% versus 40.3%) (Figure 2A). These differences may reflect divergences in TCR specificity or variation in accessory molecules on the T cell clones and/or target cells.

The MC.27.759S T cell clone expressed a TCR comprised of *TRAV21*/*TRAJ42* CDR3: CAVRLAGYGGSQGNLIF, and *TRBV12-4*/*TRBJ2.3* CDR3: CASSSQGTDTQYF, which bore little resemblance to the *TRAV38.2*/*TRAJ31* *TRBV25.1*/*TRBJ2.3* MC.7.G5 TCR we previously described (18) (Figure 2B). Curiously, both MC.27.759S and MC.7.G5 T cell clones expressed CD8-αβ (Figure 2C). This contrasted with a MAIT cell line that mainly expressed CD8-αα (Figure 2C). MR1-restricted MAIT cells are defined by high expression of CD161 (24), a phenotype that we were able to confirm (Figure 2D). Both MC.27.759S and MC.7.G5 expressed more CD161 than normal CD8-αβ^+^ T cells, but the mean intensity of fluorescent staining was less than with MAIT cells. MC.27.759S expressed over 10 times more CD161 than MC.7.G5 (Figure 2D). We explored the dependency of MC.27.759S and MC.7.G5 TCRs on CD8 expression in TCR-negative and *B2M*^–/–^ Jurkat cells lacking CD8, or expressing CD8-αα or CD8-αβ. It was essential to use *B2M^–/–^* Jurkat cells (MR1*01^+/+^) for experiments to prevent MR1 expression at the surface of these T cells, as we have previously shown that the MC.7.G5 TCR reacts to Jurkat cells (18). CD69 upregulation by Jurkat cells expressing MC.27.759S or MC.7.G5 TCRs in response to 4 different cancer cells was enhanced by the presence of CD8-αα or CD8-αβ (Figure 2E), with similar levels of CD69 seen for CD8-αα or CD8-αβ. In parallel experiments, the MAIT A-F7 TCR seemed less reliant on CD8 expression for activation toward *Mycobacterium smegmatis*–infected cells or exogenous 5-A-RU ligand at 100 μg/mL as the CD8-negative Jurkat cells responded to similar levels to those expressing CD8 (Figure 2F), perhaps due to these stimuli being strong agonists for this TCR. With Jurkat cells expressing the MC.27.759S TCR we undertook testing against more cancer cell types and assessed safety toward healthy cells. Jurkat cells expressing the MC.27.759S TCR responded to multiple cancer cell lines that naturally expressed either MR1*01 or MR1*02 but did not respond toward healthy CD14^+^ monocytes or CD19^+^ B cells from 3 donors (Figure 2G). We also tested activated B cells as target cells, as they may have increased levels of MR1 expression, alongside melanoma FM72. The B cells were activated with TLR9 ligand, as confirmed by upregulation of CD69 by the B cells (Supplemental Figure 3A). MC.27.759S activated toward the melanoma but remained inert to quiescent or activated B cells (Figure 2H). The assay included the JMA TCR (MR1 restricted and self reactive (17)) transduced Jurkat cells, which responded to the B cells, with an increased level of CD69 in the presence of activated B cells (Figure 2H).

### Sequencing of cancer-activated MR1-restricted T cells reveals a TRAJ42 bias.

**Figure 3:**
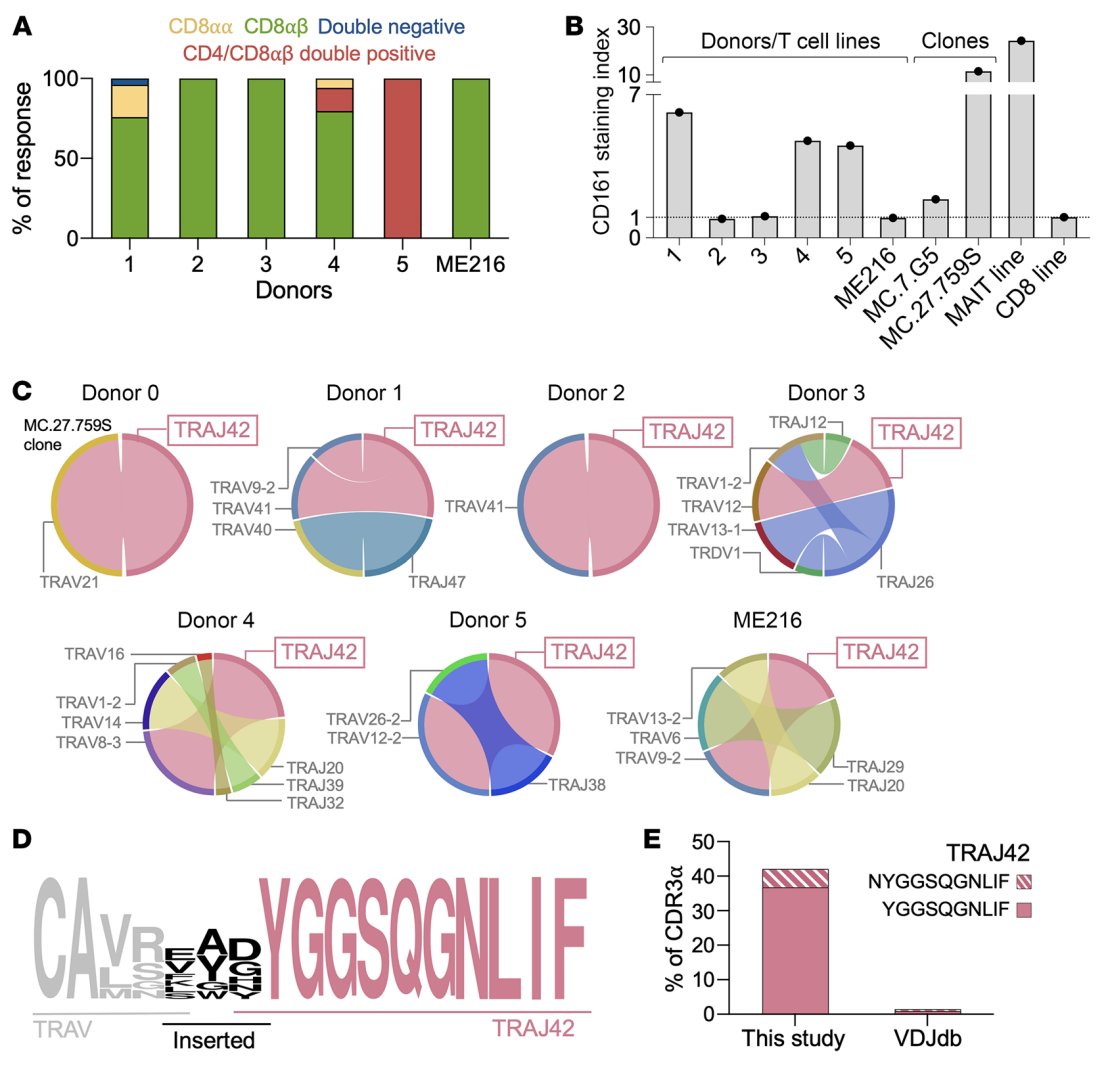
Phenotypic and clonotypic analysis of MR1-restricted cancer-reactive T cells. (**A**) CD8- and CD4-coreceptor phenotyping of MR1-reactive T cell lines from donors 0–6 and ME216. T cells from ME216 were preenriched with CD8 beads and therefore would not be CD4^+^ or CD4/CD8 double negative. (**B**) CD161 staining index (fold increase of CD161 staining relative to fluorescence minus 1 control) of 6 MR1-reactive T cell lines. MC.7.G5 and MC.27.759S clones included, as well as a MAIT and CD8^+^ T cell line. (**C**) TRAV and TRAJ gene usage of MR1-reactive T cells from 6 healthy donors and AML patient ME216. Circos plots show TRAV (V) genes on the left and TRAJ (J) genes on the right, with the size of the outer arcs corresponding to the relative frequency of the TRAV or TRAJ genes. The ribbons between the arcs represent TRAV-TRAJ pairings. (**D**) CDR3 logo plot for TCRs containing TRAJ42 with CDR3s of 17 amino acids in length from this figure and Supplemental Figure 5, giving the CAXYGGSQGNLIF motif (where X represents 5 amino acids). (**E**) TRAJ42 (preserved tyrosine with and without asparagine) summary of TCRs for donors in this figure and from the VDJdb database.

We next examined the TCR sequences expressed by T-cells from healthy donors 1–5 and AML patient 216 (ME216) that responded to the WT melanoma line. Characterization and MR1 dependency of these lines is shown in Supplemental Figure 3. Across all 6 donors the vast majority of responding cells expressed CD8-αβ (Figure 3A). Curiously, all of the cells in the donor 5 line and approximately 10% of those in the Donor 4 line expressed CD4 in addition to CD8-αβ (Figure 3A). CD161 phenotyping showed that 3 of 6 of donors had cancer-reactive T cells expressing CD161, with levels less than that of a MAIT cell line (CD161^hi^) and between those seen for clones MC.7.G5 (CD161^lo^) and MC.27.759S (CD161^medium^) (Figure 3B). Overall, of the T cell lines or clones tested, the vast majority expressed CD8-αβ and 5 of 8 (combination of T cell lines and clones) stained for CD161. TCR sequencing of the cancer-reactive T cell populations in these lines revealed recurrence of *TRAJ42* use (Figure 3C), thereby generating unusually long CDR3-α (16 or 17 amino acids) incorporating a 10 amino conserved TCR-α chain motif (Figure 3D). More specifically, the CDR3-α had 2 conserved *TRAV*-encoded amino acids (C and A), and 10 amino acids from *TRAJ42* (YGGSQGNLIF): CAXYGGSQGNLIF (X represents 4–5 amino acids). In 1 of the 8 CDR3-α (donor 3) the asparagine (N) from the TRAJ42 region had been conserved (CASMANYGGSQGNLIF).

**Figure 4:**
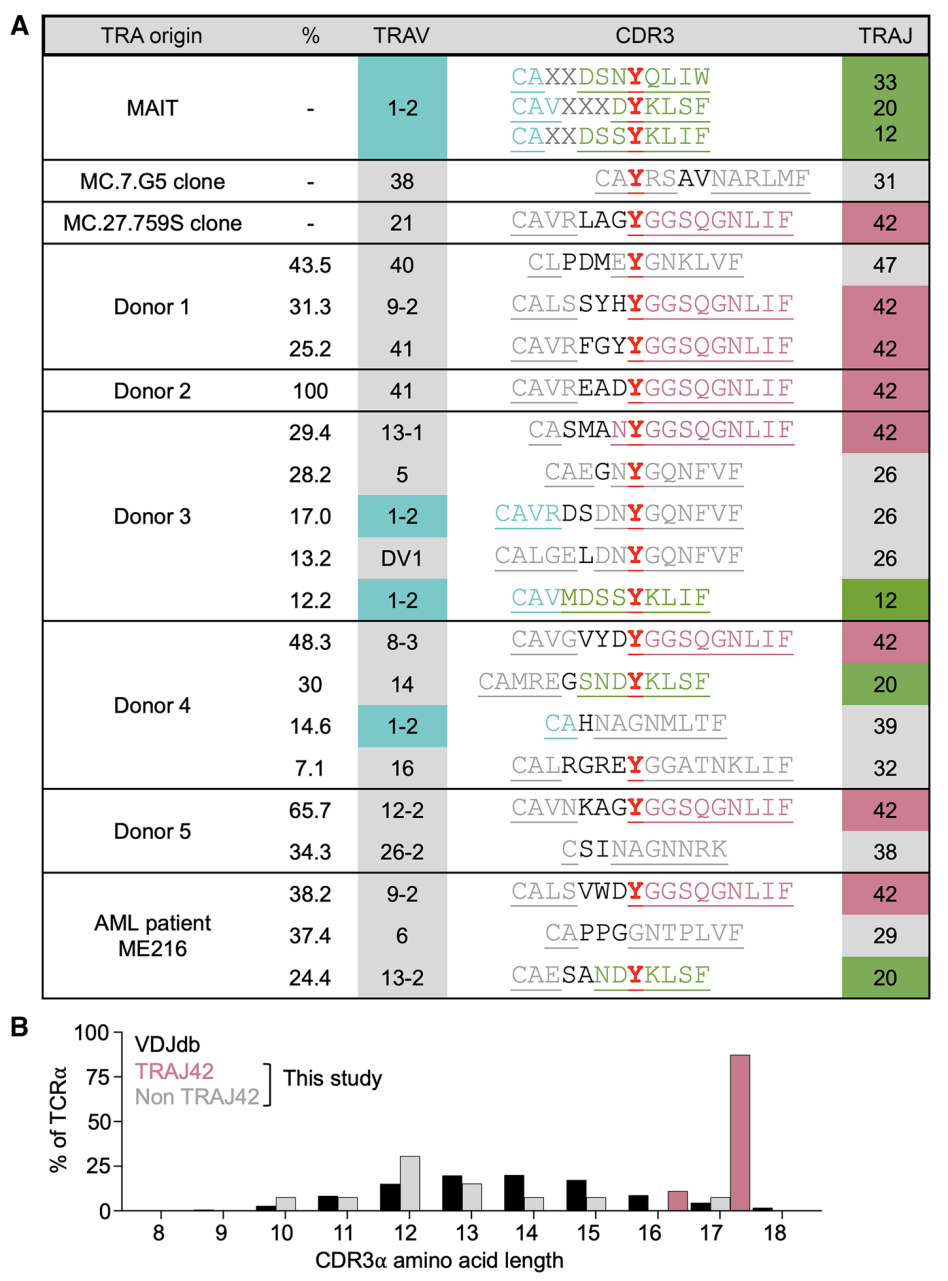
TCR-α chain usage and CDR3s of MR1-restricted cancer-activated T cells. (**A**) *TRAV*, *TRAJ*, and CDR3s of TCRs from cancer-reactive lines. Canonical MAIT T cell α chain CDR3 shown for comparison. The donors that MC.27.759S and MC.7.G5 T cell clones came from, and donors 1–5 are healthy donors. TRAV and TRAJ amino acids are underlined. (**B**) CDR3 amino acid length of antigen-defined TCRs from the VDJdb database, and *TRAJ42* or other *TRAJ* genes from donors in Figure 3.

The T cell lines from donors 1–5 and patient ME216 included a substantial population of T cells with *TRAJ42* TCRs, with this population being dominant in half of the donors with the *TRAJ42* containing TCRs making up more-than 56% of the cancer-reactive T cells. When considering that clone MC.27.759S possessed the only cancer-reactive TCR from the MR1-primed T cell lines from donor 0, then *TRAJ42* constituted over 62% of cancer reactivity across all 7 donors. *TRAJ42* was paired with 6 different *TRAV* genes within the cancer activated MR1-restricted T cell population (Figure 3C). Canonical MAIT cells use a *TRAV1-2* TCR paired with *TRAJ33*, *TRAJ20,* or *TRAJ12,* all of which provide a *TRAJ*-encoded tyrosine residue at position 95, which establishes 8 contacts with MR1 and is perfectly positioned to establish contacts with a ligand presented by in the A′ cavity of MR1 (25). It was noticeable that all *TRAJ42*-containing cancer-activated MR1-restricted TCRs maintained a similarly placed tyrosine residue (Figures 3D and Figure 4A).

In contrast to the over 42% of CDR3-α in this study that use *TRAJ42* with a preserved tyrosine, only 1.45% of TCR-α chains deposited in the VDJdb database have the same characteristic (26) (Figure 3E), and only 0.87% of CDR3s on the VDJdb database have deleted the asparagine but maintained the tyrosine from the *TRAJ42* gene segment (Figure 3E). Conservation of the tyrosine ensured that the CDR3-α of these *TRAJ42* TCRs were unusually long with 16 (*n* =1) or 17 (*n* =7) amino acids, which contrasts with the favored 13–14 amino acid length CDR3-α of all the antigen-defined TCRs within the VDJdb database (Figure 4B) and preferred 12 amino acid CDR3-α of MAIT cells (Figure 4A). The 17 amino acid CDR3-α motif CAXYGGSQGNLIF (where X represents 5 amino acids) found in 7 of 8 of the TRAJ42 TCRs we describe is present in a small minority (0.17%) of TRA chains deposited on the VDJdb database (Figure 3E).

Of the remaining cancer-activated MR1-restricted TCRs, some used the MAIT cell–preferred *TRAJ20* and *TRAJ12* to generate a similarly placed tyrosine and others used *TRAJ26*, *TRAJ47*, or *TRAJ32* to encode this tyrosine residue (Figure 4A). The TCR from MC.7.G5 differed from the other MR1-restricted TCRs we identified, in that the tyrosine was encoded by the *TRAV* gene and possessed a shorter CDR3-α of 13 amino acids (Figure 4A).

Across the T cell lines from 6 donors, 3 of 18 CDR3-α sequences did not have a central tyrosine residue. Given that up to 30% of T cells can carry 2 in frame TCR-α chains (27) it is quite likely that these sequences may not be functional in terms of MR1 recognition. This certainly seemed to be the case in Donor 5, where sequencing of the line produced a single TRBV28/TRBJ2-5 TCR-β chain but 2 TCR-α chains, with 1 containing the CDR3-α motif CAVNKAGYGGSQGNLIF (Figure 4A). Similarly, in the line from donor ME216 a dominant TCR-β chain accounted for over 86% of the sequences while the 2 dominant TCR-α chains, 1 with the conserved CALSVWDYGGSQGNLIF motif and 1 without a tyrosine, made up more-than 75% of the TCR-α chain sequences (Figure 4A).

The conservation of *TRAJ42* use across all donors suggests that these T cells respond to the same or similar MR1-restricted ligand. As with MAIT cells, there was no tight conservation in the TCR-β chain sequence across the 7 donors (Supplemental Figure 4, B and C). We also examined whether *TRAJ42* TCRs could be detected after a single priming with C1R cells overexpressing MR1*01 using 4 further donors and including previous donors 3 and 5 as positive controls. In 5 of 6 donors, one of the top 10 responding clonotypes expanded after priming with C1R-MR1*01 targets used *TRAJ42* paired with 5 different *TRAV* genes to give the same CAXYGGSQGNLIF motif, with X representing 3–5 amino acids, and the CDR3-α being 15–17 amino acids in length (Supplemental Figure 5, A and B). The dominant responding clonotype in the remaining donor used the *TRAJ12* gene that is used by some MAIT cells. We conclude that there are strong biases within the TCRs of T cell populations that respond to cancer cell targets through MR1. The most dominant bias is a conserved 10 amino acid motif that arises through use of the *TRAJ42* gene to produce usually long CDR3-α loops of 16 or 17 amino acids in length.

**Figure 5:**
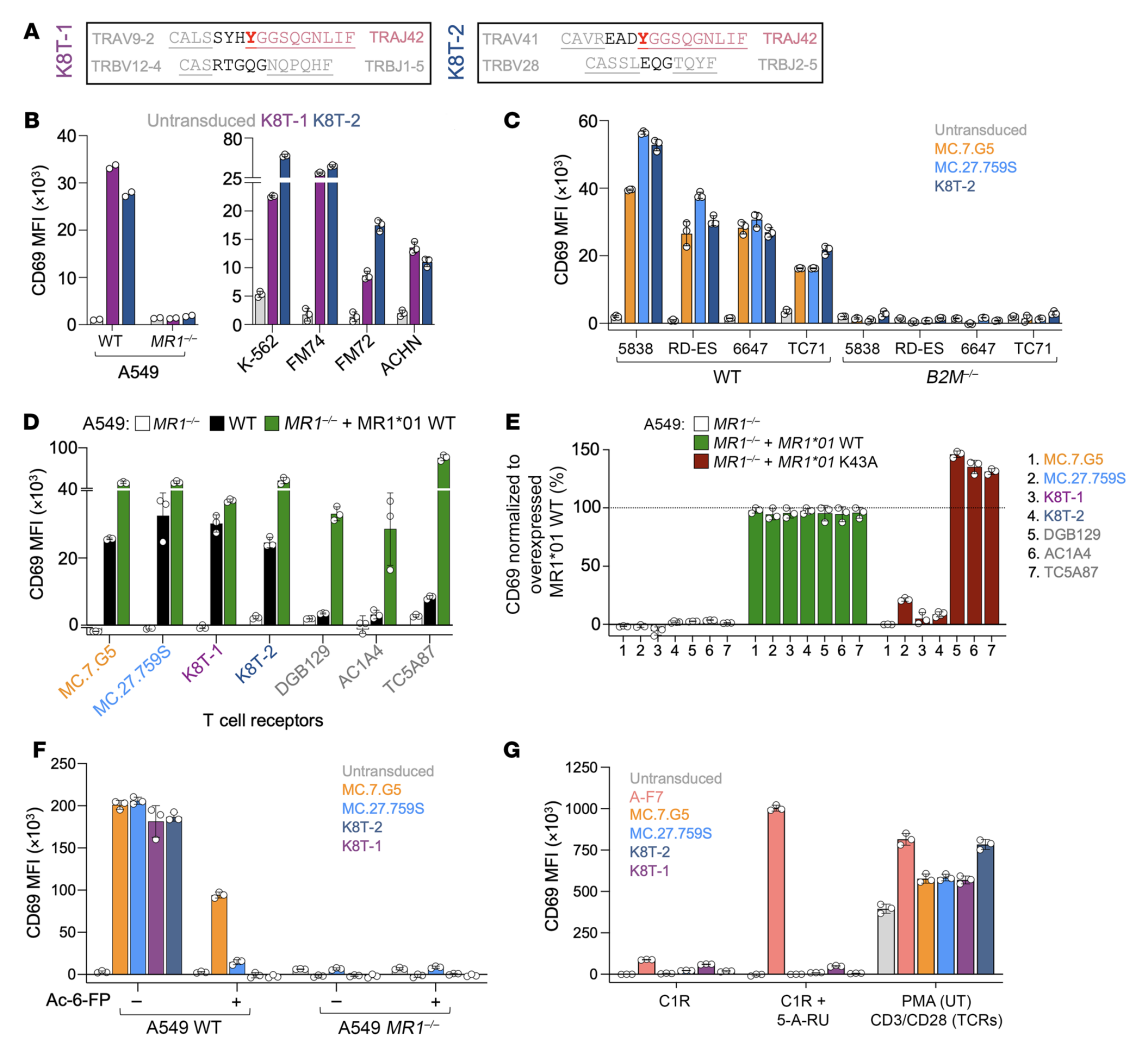
Testing MR1-restricted cancer-reactive TCRs. (**A**) Functionally paired TCRs from donors 1 and 2, named K8T-1 and K8T-2, respectively. (**B**) Left, CD69 assay (24 hours) of K8T-1 or K8T-2 TCR-transduced Jurkat cells with A549 WT and MR1-KO (^–/–^) cell lines. Duplicate conditions. Right, CD69 assay (24 hours) of K8T-1 and K8T-2 TCR-transduced Jurkat cells with MR1*01 cancer lines, including leukemia/CML (K-562), melanoma (FM74, FM72), and kidney (ACHN) cancers. Triplicate conditions with error bars depicting SD. (**C**) CD69 assay (24 hours) with MC.7.G5, MC.27.759S, and K8T-2 TCR-transduced Jurkat cells with various MR1*01 WT and *B2M^–/–^* (CRISPR/Cas9) Ewing sarcoma cell lines. Triplicate conditions with error bars depicting SD. (**D**) CD69 assay (4 hours) of MC.7.G5, MC.27.759S, K8T-1, K8T-2, DGB129, AC1A4, and TC5A87 TCR-transduced Jurkat cells with A549 cells, including WT, *MR1^–/–^* and *MR1^–/–^* with transgene for expression of scβ2M-MR1*01 WT. CD69 values of untransduced Jurkat cells have been subtracted. Triplicate conditions with error bars depicting SD. (**E**) CD69 assay (24 hours) of TCRs in **D** versus A549 *MR1^–/–^* cells, also with reexpression of scβ2M-MR1*01 WT or K43A. CD69 values of untransduced Jurkat cells has been subtracted. Data normalized to activation with scβ2M-MR1*01 WT. Triplicate conditions with error bars depicting SD. The K8T-2 TCR was performed in a separate assay with quadruplicate conditions, error bars show SD. (**F**) CD69 assay (24 hours) of MC.7.G5, MC.27.759S, K8T-1 and K8T-2 TCR-transduced Jurkat cells with A549 cell ± MR1 ± 10 μg of Ac-6-FP. (**G**) CD69 assay (24 hours) of MC.7.G5, MC.27.759S, K8T-1, and K8T-2 TCRs in Jurkat cells with C1R cells ± 10 μg of 5-A-RU. MAIT TCR A-F7 used as a positive control for 5-A-RU recognition. PMA (UT Jurkat cells) or CD3/CD28 (TCR transduced) as a positive control for CD69 upregulation.

We next tested paired TCRs with this *TRAJ42* motif from Donors 1 and 2 to demonstrate that they were functional. Sequencing of the MR1-restricted, cancer-reactive T cells from Donor 1 using a microfluidics-based single-cell sequencing platform revealed 3 paired TCRs, with 2 of the α chains being paired with 1 TRB chain (Supplemental Figure 4A). We elected to work on the dominant TRAJ42-containing TRA chain that paired with a unique TRB chain, giving the K8T-1 TCR (TRAV9-2/TRAJ42 CDR3-α CALSSYHYGGSQGNLIF and TRBV12-4/TRBJ1-5 CDR3-β CASRTGQGNQPQHF; conserved 10 amino acid *TRAJ42*-encoded motif in underlined text here and below). Donor 2 had 1 TRA and 1 TRB chain, named the K8T-2 TCR (TRAV41/TRAJ42 CDR3-α CAVREADYGGSQGNLIF and TRBV28/TRBJ2-5 CDR3-β CASSLEQGTQYF) (Figure 5A). Both K8T-1 and K8T-2 TCRs were shown to recognize A549 WT cells but not the corresponding CRISPR/Cas9 MR1-KO cell line (Figure 5B). Jurkat cells expressing K8T-1 or K8T-2 TCRs responded to K-562, FM74, FM72, MOLT-3, Kasumi-3, SiHa, and ACHN cancer cells (Figure 5B and Supplemental Figure 5C).

We took the opportunity to test MC.7.G5, MC.27.759S, K8T-1, and K8T-2 TCRs in the same assays using transduced *B2M*^–/–^ Jurkat cells, first against Ewing sarcoma cancer cell lines ES-5838, RD-ES, 6647, and TC71 (all MR1*01^+/+^), which were recognized to similar levels in a β2M-dependent manner by MC.7.G5, MC.27.759S, and K8T-2 (Figure 5C). We also compared our TCRs (MC.7.G5, MC.27.759S, K8T-1, and K8T-2) to non-MAIT MR1-restricted TCRs from other studies (DGB129, TC5A87, and ACA14) that had been reported to only recognize cells overexpressing MR1 and exhibit no dependency on the K43 residue of MR1 for activation. MC.7.G5 MC.27.759S, K8T-1, and K8T-2 TCRs reacted toward A549 WT cells in a MR1-dependent manner, whereas DGB129, TC5A87, and ACA14 showed minimal reactivity toward A549 cells but they did respond to A549 cells overexpressing scβ2M-MR1*01 (Figure 5D). We also generated and tested A549 MR1-KO cells with overexpressed scβ2M-MR1*01 with a K43A substitution. Cell surface expression of MR1*01 was enhanced in the presence of K43A compared with K43, which has been reported previously due to neutralization of the positively charged K43, which retains MR1 in the endoplasmic reticulum (Supplemental Figure 5D) (28). DGB129, TC5A87, and ACA14 reacted maximally to the A549 cells overexpressing scβ2M-MR1*01 K43A (Figure 5E), most likely due to the enhanced cell surface expression of scβ2M-MR1*01 K43A relative to scβ2M-MR1*01 without this mutation. In contrast, MC.7.G5, MC.27.759S, K8T-1, and K8T-2 exhibited much reduced activation toward targets overexpressing MR1*01 K43A, with MC.7.G5 being the most dependent on K43, followed by K8T-1 and K8T-2, then MC.27.759S (Figure 5E). Finally, we tested MC.7.G5, MC.27.759S, K8T-1, and K8T-2 with the known MR1 ligands Ac-6-FP and 5-A-RU (to produce 5-OP-RU). Ac-6-FP (10 μg/mL) partially blocked MC.7.G5 recognition of A549 WT cells, whereas reactivity of the other TCRs was substantially (MC.27.759S) or totally (K8T-1 and K8T-2) reduced (Figure 5F). None of the cancer-reactive TCRs tested reacted toward MAIT ligand precursor 5-A-RU (10 μg/mL), whereas the MAIT cell TCR A-F7 in the same assay showed robust recognition of 5-A-RU (Figure 5G).

### MR1-restricted cancer-activated T cells are not MR1 allomorph-specific and do not react to healthy cells.

A recent prepublication claimed that MR1 allomorphs drive the specificity of MR1-restricted TCRs and asserted that the MC.7.G5 TCR only responded to targets expressing MR1*04 (29). This claim puzzled us, as many of the cancer lines used in our 2020 publication (18) did not express this allele (cell lines that the MC.7.G5 TCR responded to are denoted in purple in Supplemental Figure 2). In addition, 23 of the 25 cancer cell targets tested during this study and recognized by MC.27.759S, MC.7.G5, K8T-1, or K8T-2 do not express MR1*04 (summarized in Supplemental Figure 2). Indeed, MC.7.G5 and MC.27.759S clones killed MR1*01-homozygous cancer cells ES-5838, RD-ES, and FM72 to levels seen with A549 cells (Figure 2A), which are MR1*01/*04, so our results do not support assertions that the MC.7.G5 TCR, or other TCRs, target cells via an allogenic reaction with the rare MR1*04 variant carried by approximately 0.8% of the population (15).

**Figure 6:**
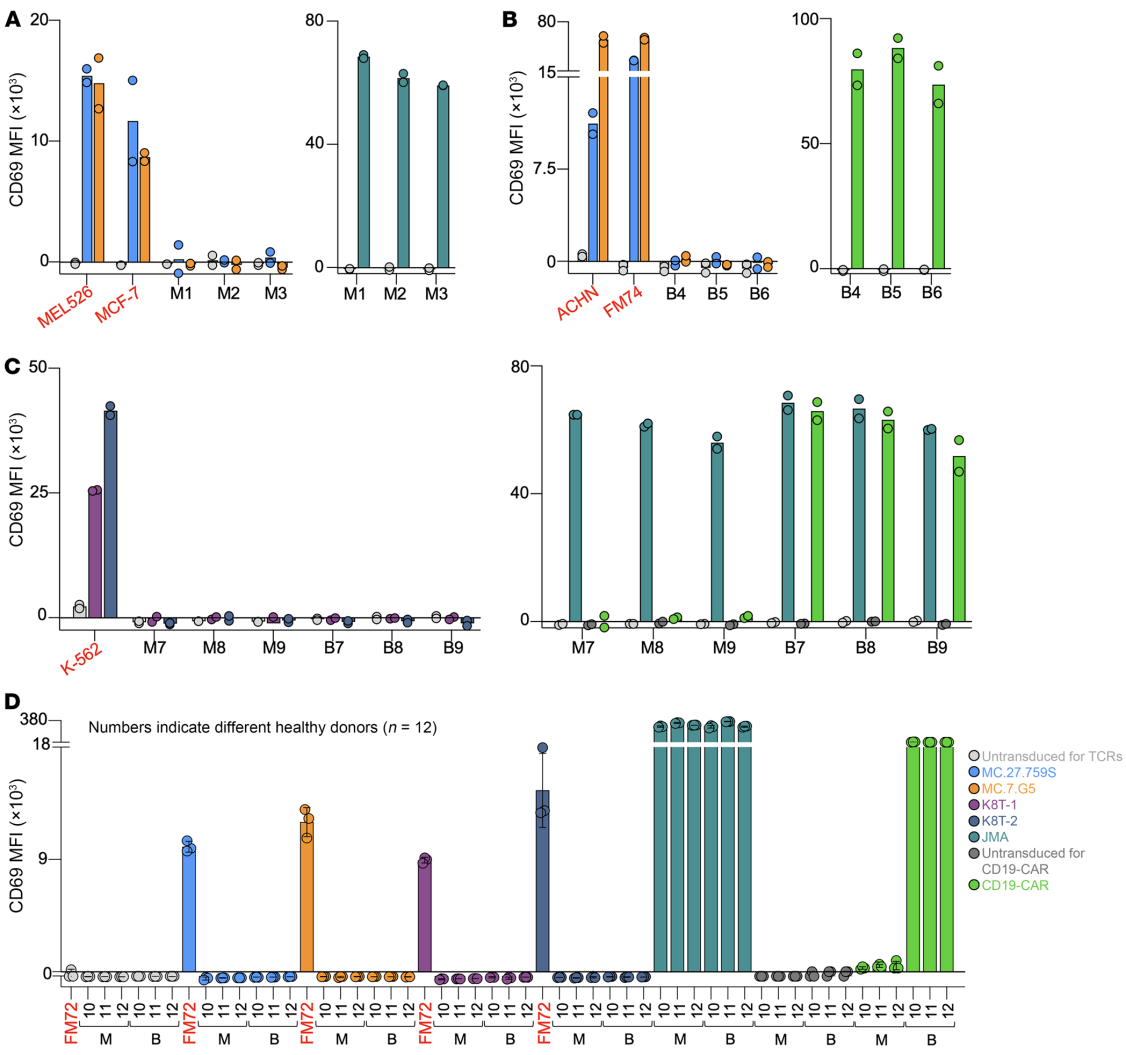
MR1-restricted TCRs do not react to healthy cells and are cancer specific. (**A**) CD69 assay (24 hours) of MC.27.759S and MC.7.G5 TCR-transduced Jurkat cells with cancer cell lines MEL526 (melanoma, MR1*01/*02) and MCF-7 (breast, MR1*01/*02), and purified (CD14^+^) monocytes (M). MR1T TCR JMA in Jurkat cells used as a positive control for monocyte recognition. Duplicate conditions. (**B**) CD69 assay (24 hours) of MC.27.759S and MC.7.G5 TCR-transduced Jurkat cells with cancer cell lines ACHN (kidney, MR1*01) and FM74 (melanoma, MR1*01), and purified (CD19^+^) B cells. CD19-chimeric antigen receptor (CAR) expressed in Jurkat cells were used as a positive control for B cell recognition. Duplicate conditions. (**C**) CD69 assay (24 hours) of K8T-1 and K8T-2 TCR-transduced Jurkat cells cancer cell line K-562 (leukemia/CML, MR1*01), and purified monocytes (CD14^+^) and B cells (CD19^+^). JMA TCR and CD19-CAR in Jurkat cells were used as positive controls for healthy cell recognition. Duplicate conditions. (**D**) Repeated CD69 assay (24 hours, conditions in triplicate with error bars depicting SD) using the above TCRs and CD19-CAR with monocytes (M) and B cells (B) from 3 healthy donors and cancer cell line FM72 (melanoma, MR1*01).

It was also important to extend the safety testing already performed with the MC.7.G5 (18) and MC.27.759S (Figure 2, F and G) TCRs. We assessed whether MC.7.G5, MC.27.759S, K8T-1, and K8T-2 TCRs were cancer-specific by testing them with healthy cells. Previous experiments examining this aspect have used healthy monocytes and B cells as these cell types express the highest natural levels of MR1 (29). Jurkat cells expressing MC.7.G5, MC.27.759S, K8T-1, or K8T-2 TCRs responded to MR1*01 homozygous or MR1*01/*02 cancer cell lines but, collectively, did not respond to monocytes or B cells from sixteen different healthy donors (Figure 2, F and G and Figure 6). In contrast, Jurkat cells expressing the MR1-restricted JMA TCR (17) responded to the monocytes from 9 of 9 donors and B cells from 7 of 7 donors, and Jurkat cells expressing a CD19-CAR responded to B cells from 9 of 9 donors (Figure 2, F and G and Figure 6). We conclude that the MC.7.G5, MC.27.759S, K8T-1, and K8T-2 TCRs do not respond to healthy cell lines, including monocytes and B cells, which are claimed to express high natural levels of MR1 (29).

### TCR replacement vastly improves the sensitivity of T cells transduced with MR1-restricted TCRs.

**Figure 7:**
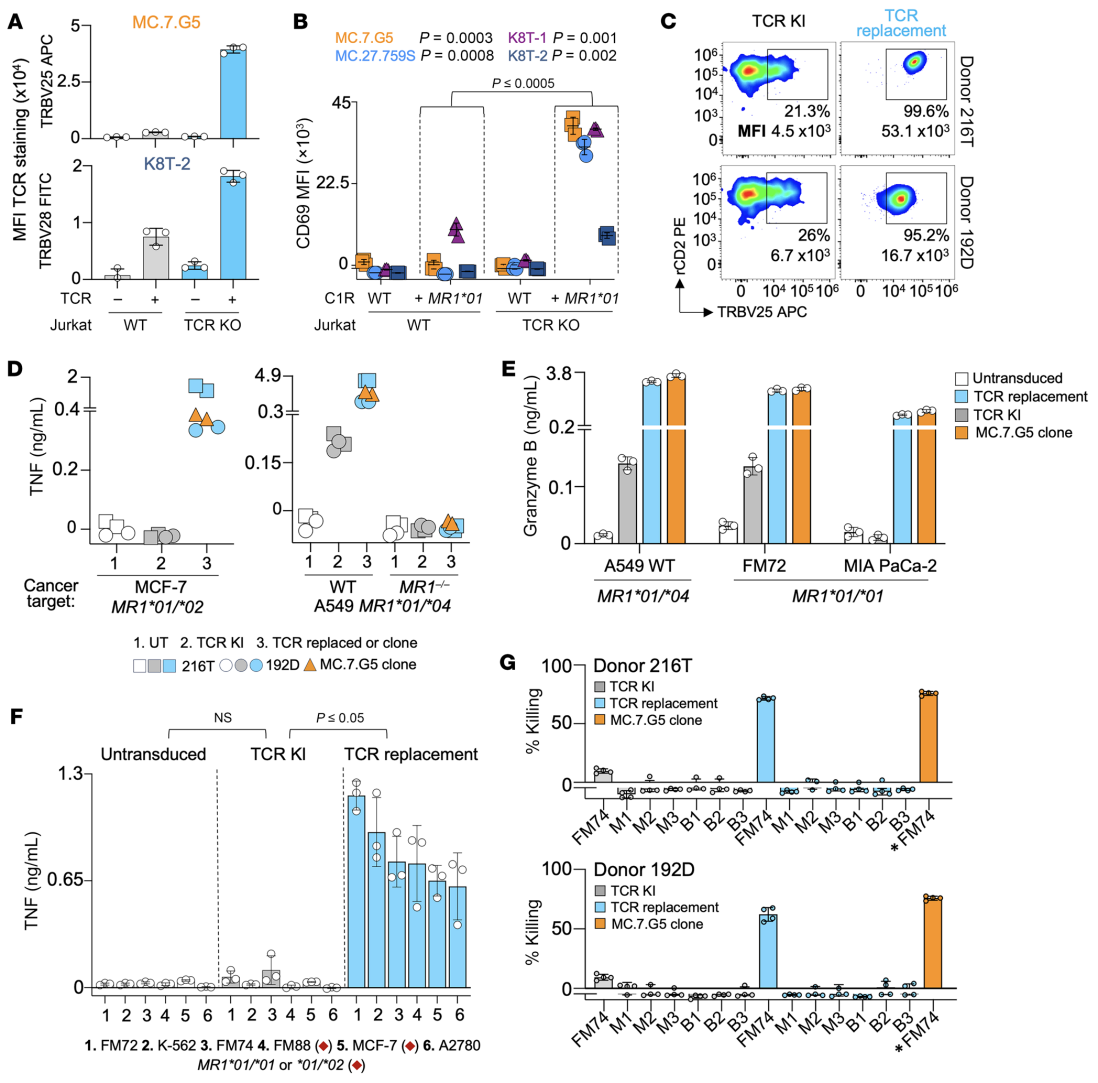
Cancer reactivity requires TCR replacement. (**A**) WT or TCR-KO E6.1 Jurkat cells with MC.7.G5 or K8T-2 TCRs stained with TRBV25 or TRBV28 antibodies, respectively. Conditions in triplicate, error bars depict SD. (**B**) WT or TCR-KO E6.1 Jurkat cells with MC.7.G5, MC.27.759S, K8T-1, or K8T-2 TCRs in a CD69 assay (24 hours) with C1R WT or overexpressed MR1*01. Performed over 3 assays: MC.7.G5, MC.27.759S, and K8T-1/K8T-2. Conditions in triplicate, error bars depict SD. Statistics for WT and TCR-KO Jurkat cells versus C1R + MR1*01. Individual *P* values for a Shapiro-Wilk normality and paired 2-tailed t test. Collective *P* value for a Wilcoxon signed-rank test. (**C**) Purified CD8^+^ T cells from 2 healthy donors (216T and 192D) transduced with MC.7.G5 TCR, comparing KI with TCR replacement. Staining with rCD2 (TCR comarker) and TRBV25 for MC.7.G5 TCR expression. (**D**) Donors 216T and 192D CD8^+^ T cells, either untransduced, MC.7.G5 TCR KI or TCR replaced in an overnight activation assays with cancer cells, followed by TNF ELISA. MC.7.G5 clone, included for comparison. Duplicate conditions. (**E**) Donor 216T CD8^+^ T cells, either untransduced, MC.7.G5 TCR KI or TCR replaced in an overnight activation assay with cancer cells, followed by Granzyme B ELISA. Triplicate conditions, error bars depict SD. (**F**) Donor 216T CD8^+^ T cells, either untransduced, MC.7.G5 TCR KI or TCR replaced in an overnight activation assay versus MR1*01 or MR1*01/*02 cancer cells, followed by TNF ELISA. Triplicate conditions, with error bars depicting SD. *P* value for a multivariate permutation test for paired comparison. (**G**) Donor 216T CD8^+^ T cells, either untransduced, MC.7.G5 TCR KI or TCR replaced in an overnight flow cytometry–based killing assay at a 1:1 ratio with cancer cells FM72, or PBMCs from 3 healthy donors. CD14 and CD19 antibodies used to identify monocytes (M) and B cells (B) during analysis. MC.7.G5 clone data repeated (*) on each graph for comparison. Cancer cells include melanoma (FM72, MEL624, MEL526, FM3, FM88, and FM74), kidney (ACHN), pancreatic (BxPC-3 and MIA PaCa-2), leukemia/CML (K-562), breast (MCF-7), ovarian (A2780), and cervical (SiHa) cancers.

The results of a previous study (29) contrasted with our own by finding that the MC.7.G5 and MC.27.759S TCRs did not respond to MR1*01 homozygous and MR1*01/*02 cancer cell lines, prompting us to consider what had caused this disparity. Dukes and colleagues (29) used TCR transduction in their study, whereas we now routinely use TCR replacement, as the endogenous TCR can reduce the ligand-sensitivity of transduced T cells by several orders of magnitude (23). We hypothesized that our use of T cell clones and TCR replacement might explain the differences in results between the laboratories’ results (18, 29). We used TCR transduction with and without knockout of the endogenous *TRBC1* gene to express the MC.7.G5 or K8T-2 TCRs in Jurkat cells prior to staining with antibody specific for TRBV25 or TRBV28, respectively (Figure 7A). Knockout of the Jurkat cell endogenous TCR-β chain gave an increase in expression for the MC.7.G5 TCR of 1311.3% and 141.8% for the K8T-2 TCR (Figure 7A). We did not undertake the TRBV staining experiments with MC.27.759S and K8T-1 as they express the same TRBV as the Jurkat cell line, however, we did test all 4 TCRs expressed in Jurkat WT and *TRBC1*-KO cells in activation assays versus C1R cells overexpressing MR1*01 (Figure 7B). CD69 upregulation by MC.7.G5 (*P* = 0.0003), MC.27.759S (*P* = 0.0008), K8T-1 (*P* = 0.001), and K8T-2 (*P* = 0.002) TCRs was at its maximum in the absence of endogenous *TRBC1* (collective *P* value of 0.00049), with only K8T-1 showing some reactivity in WT Jurkat cells toward the C1R cells overexpressing MR1*01 in the absence of *TRBC1* KO (Figure 7B). Jurkat cells expressing the K8T-2 TCR were the least reactive to C1R cells overexpressing MR1*01 (Figure 7B).

We also used TCR replacement in parallel with TCR transduction without disruption of the endogenous TCR-β chain (TCR knockin; TCR KI) to express the MC.7.G5 TCR in purified CD8^+^ T cells from 2 healthy donors. For TCR KI, only 21% and 26% of CD8^+^ T cells expressing the rat CD2 transduction marker stained with TRBV25 antibody, which is a similar level of transduction to that observed by Dukes and colleagues using a partially murinized TCR construct (29). The low frequency of staining with TRBV25 antibody following TCR transduction with the MC.7.G5 TCR contrasted with the 99.6% and 95.2% staining observed with TCR replacement. In addition to a greater frequency of staining there was a noticeable improvement in the intensity (11.8 and 2.5-fold in Donors 216T and 192D, respectively) of staining with TCR replacement (Figure 7C). The wide difference in TCR expression between studies could be crucial in explaining why results differ, as the level of TCR expression is probably the single most important contributor to the antigen sensitivity of a T cell (23).

We compared how Donor 216T and 192D CD8^+^ T cells transduced with the MC.7.G5 TCR ± TCR replacement and the MC.7.G5 T cell clone recognized the MR1*01/*02 breast cancer cell line MCF-7 using TNF as a readout. Only the MC.7.G5 T cell clone and CD8^+^ T cells expressing the MC.7.G5 TCR via TCR replacement in both donors showed a response to the MCF-7 breast cancer line (Figure 7D). In agreement with the findings of Cornforth et al (29) we were able to show that TCR-transduced T cells were capable of recognizing the MR1*01/*04 target cell A549, albeit substantially lower than the activation (TNF or Granzyme B) seen for TCR-replaced TCR-T cells and the MC.7.G5 clone (Figure 7, D and E). Additionally, the TCR-KI T cells released granzyme B when incubated with melanoma FM72, which is a very good target of the MC.7.G5 TCR and MR1*01 homozygous, but also at very reduced levels of activation compared with the TCR-replaced T cells (Figure 7E). In the same assay, release of granzyme B in response to pancreatic cancer MIA PaCa-2, which is a weaker target of MC.7.G5, was fully reliant on TCR replacement (Figure 7E). Recognition of MR1*01/*04 A549 cells suggests the rare MR1*04 variant might be a more sensitive TCR ligand for the MC.7.G5 TCR than MR1*01 or MR1*02 or indicate that the MR1*04 protein might present the cancer ligand better than other MR1 variants.

Next, we tested CD8^+^ T cells from Donor 216T expressing the MC.7.G5 TCR against a wider panel of MR1*01^+/+^ or MR1*01/*02 cancer cells (Figure 2F and Supplemental Figure 5, E and F). Ablation of *TRBC1/2* genes led to significantly higher amounts of TNF in response to the cancer cells (*P* =0.032 Figure 7F and *P* = 0.001 Supplemental Figure 5F), where there was no significant response (*P* = 0.13) from donor cells that had been transduced with the MC.7.G5 TCR without *TRBC* gene ablation (Figure 7F). Similar results were seen from killing assays using CD8^+^ T cells from Donors 216T and 192D; MC.7.G5 TCR-replaced CD8^+^ T cells and the MC.7.G5 T cell clone showed good killing of MR1*01 homozygous melanoma line FM74 at an effector-to-target ratio of 1:1, while CD8^+^ T cells from both donors transduced with the MC.7.G5 TCR without KO of the endogenous TCR-β chain showed little killing of these cancer targets (Figure 7G). Importantly, all of the T cells tested remained inert to the monocytes and B cells from 3 healthy donors (Figure 7G).

In summary, we were able to reproduce the results of Dukes and colleagues (29) and those of our original study with the MC.7.G5 TCR clone and TCR (18) and showed that the large difference in TCR transduction frequency and expression between the 2 studies (which results in a major difference in T cell sensitivity) provided a likely explanation for why the results and conclusions differed.

## Discussion

We recently described an MR1-restricted T cell clone that responded to a wide range of cancer cell lines while remaining inert to healthy cells (18). Here, we succeeded in generating similar cancer-activated MR1-restricted T cells from all donors. These T cells were able to respond to WT levels of MR1 on a wide range of cancer cell lines from many different origins but remained inert to healthy CD14^+^ monocytes and B cells from multiple individuals. The TCR from MC.7.G5 T cell clone and the TCR from a similar clone, MC.27.759S, failed to confer a response to *MR1*-KO cancer lines and showed high dependence on the lysine residue at position 43 in MR1, which is known to form a Schiff base with the known MR1-presented bacterial ligands (19). Both MC.7.G5 and MC.27.759S T cell clones expressed CD8-αβ, and CD8 expression in Jurkat cells expressing these and other MR1-restricted TCRs enhanced the response to cancer targets. CD8-αβ is generally expressed by HLA class I–restricted T cells where it augments TCR-mediated signal transduction by recruiting the lymphocyte-specific protein kinase (Lck) to the intracellular side of the TCR/CD3 complex to ensure full phosphorylation of immunoreceptor tyrosine kinase motifs (ITAMs) on the cytoplasmic CD3 domains, especially CD3-ζ (3, 30). CD8 also acts to stabilize TCR binding to peptide MHC at the T cell surface, extend the range of ligands recognized and control the deployment of conventional CD8^+^ T cell effector functions (31–35). CD8-αβ is a better coreceptor for HLA-I–restricted T cells than CD8-αα (36, 37). Unconventional T cells like intraepithelial lymphocytes (IELs), iNKT cells, γδ T cells,and a proportion of MAIT cells express CD8-αα (38, 39). Indeed, approximately 90% of all T cells in human blood that express CD8-αα are MAIT cells (40). The MC.7.G5 and MC.27.759S clones also expressed the NK cell marker CD161 that we did not see widely on CD8^+^ T cells, with expression being far higher on the MC.27.759S clone than MC.7.G5 (MFI 39,761 versus 3711) but less than on a MAIT cell line in a parallel experiment (MFI 49,606). Of the MR1-restricted cancer-reactive T cell lines that were generated, 3 of 6 expressed CD161, with levels residing between that of MC.7.G5 and MC.27.759S. CD161 is a C-type lectin-like receptor that is highly expressed by most NK cells and defines a functionally distinct subset of proinflammatory NK cells that retain the ability to respond to innate cytokines; a property that is shared by CD161^+^CD8^+^ T cells (41). The CD161^+^CD8^+^ T cell subset is dominated by MAIT cells and can be specifically activated by IL-12 and IL-18 in a TCR-independent manner (42). CD161 defines a transcriptional and functional phenotype across distinct human T cell lineages (43). Our results indicate that the MR1-restricted cancer-activated T cells we describe may also reside in this CD161^+^CD8^+^ T cell population and might be specifically demarked from the majority of CD161^+^CD8^+^ T cells by their lack of *TRAV1-2* and CD8-αα expression (*TRAV1-2*^neg^, CD8αβ^+^, CD161^lo/med^), although further work will be required to confirm this.

TCR sequencing of MR1-restricted cancer-activated T cells showed that the majority of these cells shared a conserved 10-amino acid–long TCR-α chain motif as a result of *TRAJ42* use. Across our study, *TRAJ42* was paired with 10 different TRAV genes, giving an overall motif of CAXYGGSQGNLIF (where X represents 3–5 amino acids), with a near central tyrosine residue. A similar tyrosine was present in 24 of 27 of the TCR-α chains we describe where it was encoded by *TRAJ26*, *TRAJ29*, *TRAJ32*, *TRAJ38*, *TRAJ39*, *TRAJ47*, and *TRAJ12* or *TRAJ20*, which are sometimes used by MAIT cell TCRs to provide a similarly placed tyrosine residue, which makes important contacts with MR1 and its bacterially derived cargo (25). The bias toward *TRAJ42* use and conservation of a semi-invariant motif makes it tempting to speculate that the cells we describe across multiple donors likely exhibit limited antigen diversity and might bind to an identical MR1-bound cargo at the cancer cell surface. No obvious patterns were seen within the TCR-β chain of MR1-restricted cancer-activated T cells.

We next addressed claims made in a recent study (29), which suggested that recognition by cancer-reactive MR1-restricted TCRs, including MC.7.G5 and MC.27.759S, was restricted to the MR1*04 variant and was not cancer-specific. This study also asserted that Jurkat cells expressing the MC.7.G5 TCR only responded to MR1*01 expressed on targets at supraphysiologic expression levels (29). These claims did not align with our previously published findings that demonstrated recognition of natural levels of MR1 on multiple MR1*01^+/+^ and MR1*01/*02 cancer cell lines, as highlighted in purple in Supplemental Figure 2 (18). Indeed, we used WT Jurkat cells (MR1*01^+/+^) alongside *MR1^–/–^* Jurkat cells in the murine model in our previous study where only the former were cleared from mice infused with MC.7.G5 T cells (18). The work presented here strongly supports our earlier work and refutes 2 claims made by Cornforth et al. (29). First, multiple lines of evidence show that recognition of target cells by the MC.7.G5 TCR is not restricted to MR1*04 but extends to other far more common MR1 variants. Only 2 of the many targeted cell lines used in this study express MR1*04 (Supplemental Figure 2). Cancer cell lines that were not MR1*04^+^, including Jurkat cells (MR1*01^+/+^), as used in the in vivo model in our previous study, were recognized well (18). Other researchers have also described similar cancer-reactive MR1-restricted T cells that target non-MR1*04^+^ targets (44, 45). A recent study by Niekens et al. repeated our findings showing that TCR-T cells expressing the MC.7.G5 TCR kill the adult cancer lines K-562 and Jurkat (44), which do not express MR1*04 (18, 44). This study further showed that reactivity of MC.7.G5 TCR-T cells extended to a wide range of pediatric cancers, which included leukemia and glioma (44). Cancer recognition encompassed patient-derived organoid and patient-derived xenograft samples (44). MR1 allotype testing of recognized cancer lines led the authors to concluded that MC.7.G5 TCR-expressing T cells exhibited on-target cancer-reactivity across several *MR1* allelic variants (44). Kishi and colleagues (45) described expansions of HLA-agnostic T cells in the TILs of 2 patients with breast cancer in Japan who responded to MCF-7 breast cancer cells (MR1*01/*02) but not MR1-KO MCF-7 cells. Cell targeting did not require the presence of MR1*04. Furthermore, the expansion of these breast cancer–reactive, MR1-restricted T cells within breast cancer TIL without described pathology indicates that these cells are safe in vivo. A recent follow up study confirmed that the most responsive of these T cell clones from patient number 10, clone 10-59, expressed a *TRAV26-1/TRAJ42* TCR that has a CDR3-α including the tyrosine-containing 10 amino–acid sequence, YGGSQGNLIF, which we describe here to mean that 2 independent laboratories have now found similar MR1-restricted, cancer-activated invariant T cells (CAITs) (46). Second, we show that the MC.7.G5 TCR is cancer-specific, as it failed to respond to any of the many healthy cell lines tested in this study or our previous study (18). Importantly, we show that use of TCR transduction instead of TCR replacement (23) resulted in only a minority of transduced primary CD8^+^ T cells staining for the transduced MC.7.G5 TCR-β chain and that this minimal staining was of lower intensity. We have previously demonstrated that the antigen-sensitivity of TCR-transduced T cells in the absence of measures to disrupt endogenous *TRBC1* and *TRBC2* genes can be diminished by as much as 3 orders of magnitude (23). This was highly apparent with the MC.7.G5, MC.27.759S, K8T-1, and K8T-2 TCRs expressed in normal Jurkat cells, where CD69 upregulation of cells transduced without CRISPR KO of the endogenous Jurkat *TRBC1* genes was reduced (*P* ≤ 0.0005). The difference of not using TCR replacement was also apparent using primary CD8^+^ T cells, as such cells were unable to respond to cancer targets while TCR-replaced CD8^+^ T cells gave robust responses in parallel (*P* ≤ 0.05 and 0.001, depending on assay). Our results showing the insensitivity of MC.7.G5 TCR-transduced CD8^+^ T cell products, where steps are not taken to ablate the endogenous TCRs (18, 23), explain the differences observed between our study and those of Dukes and colleagues (29) and further serve to highlight the importance of preventing TCR competition for cell surface expression within individual T cells.

The remaining discussion point concerns why the rare MR1*04 variant appears to be a better ligand for the MC.7.G5 TCR and other cancer-activated MR1-restricted TCRs. The key difference between MR1*04 and all the other *MR1* allomorphs that are present in over 99% of the population is the substitution of the arginine at position 9 for a histidine, the only polymorphism that could affect ligand binding and TCR recognition (Supplemental Figure 1). Rossjohn and colleagues recently described an individual with immunodeficiency who was homozygous for the R9H variant of MR1 (16). This individual was found to be completely lacking in the important MAIT cell subset, a deficiency explained by the fact that the MR1*04 protein was incapable of presenting the MAIT cell ligand 5-(2-oxopropylideneamino)-6-D-ribitylaminouracil (5-OP-RU) (16). We previously demonstrated that mycobacterial infection or incubation with Ac-6-FP blocked target recognition by the MC.7.G5 TCR (18), and, in this study, Ac-6-FP blocked target recognition by MC.27.759S, K8T-1, and K8T-2 TCRs. This is presumably due to competition for K43 in MR1. The fact that MR1*04 has a ligand binding site difference that precludes presentation of MAIT cell ligands might mean that this variant binds to cancer ligands better than other MR1 variants or that these ligands are more likely to be presented in the absence of competition from standard self and bacterial ligands that are unable to bind to MR1*04. Further work will be required to determine whether the enhanced recognition of MR1*04^+^ target cells is due to altered ligand presentation, altered TCR interactions, or both. Although our results with MC.7.G5 TCR-transduced CD8^+^ T cells show some preferential recognition of MR1*04^+^ cancer cells, we were unable to reproduce the large differences in recognition observed in a previous study (29). We have previously observed and demonstrated (47, 48), on multiple occasions across multiple systems, that it is impossible to culture T cell–reactive primary T cells as presumably they succumb to T cell–versus–T cell fratricide. While it is possible to grow HLA A*02:01^neg^ CD8^+^ or CD4^+^ T cells bearing supraphysiologic affinity HLA A*02:01-restricted TCRs, these T cells react to an HLA A*02:01^+^ target cells without the need for cognate antigen as they presumably start to recognize some self antigens with sufficient affinity to activate (48). HLA A*02:01^+^ CD8^+^ or CD4^+^ T cells bearing these same TCRs will not grow in parallel (47, 48). By extension, we would expect that MR1*04^+^ T cells would not thrive when transduced with the MC.7.G5 TCR if it were alloreactive, as suggested by Dukes and colleagues (29). However, we have not observed such alloreactivity. When transduced with the MC.7.G5 TCR, CD8^+^ T cells from patients with melanoma referred to as MM909.11 and MM909.24 were able to respond to the patient-autologous melanoma lines (18). Patient MM909.24 is MR1*01/*04, meaning that the autologous T cells from this patient that we transduced for these experiments in our previous study (18) were MR1*04^+^. We had no issue culturing these cells and they showed no sign of being autoreactive. Consequently, the experiments we have already performed do not suggest that there is any hint of alloreactivity toward healthy MR1*04^+^ target cells.

In summary, we were able to grow MC.7.G5-like T cells from all healthy donors tested. These cells recognized a wide range of cancer cell lines via MR1 regardless of what MR1 allomorph they expressed. Recognition of cancer cells showed dependency on the K43 residue. This dependency suggests that the cancer-associated ligand(s) might form a Schiff base with this residue, as is known to occur with known microbial ligands (19), or, alternatively, might reflect changes in the MR1 antigen-binding pocket in the absence of the positively charged lysine residue. As previously reported (18), recognition by MC.7.G5 T cells and MC.7.G5-like T cells was cancer-specific, as these T cells responded to a wide range of cancer cell targets while remaining inert to healthy cell lines, including healthy B cells and monocytes. Many of the MC.7.G5-like T cells we describe in our study were CD161^lo/med^CD8-αβ^+^. Strikingly, most of the MC.7.G5-like T cells we describe incorporated a conserved CAXYGGSQGNLIF amino acid motif, where X represents 3–5 amino acids, in their unusually long CDR3-α. The conservation of this public-like TCR motif across all but 1 donor we studied and the unusual, near ubiquitous, expression of the CD8-αβ isoform defines CAITs as a new T cell subset and suggests that they respond to a common MR1-restricted ligand on the surface of cancer cells. Further work will be required to determine what this ligand is and why it appears to be preferentially presented by cancer cells. Our recent methodology for identifying novel, stabilized Schiff base–bound MR1 ligands at the cell surface via crosslinking mass spectrometry (49) will hopefully aid the discovery of new, natural MR1 ligands, including those recognized by CAITs and other cancer-activated MR1-restricted T cells.

## Methods

### Sex as a biological variable.

Sex was not considered as a biological variable in this study.

All procedures are described in detail in the Supplemental Methods.

### Statistics.

Unless stated otherwise, all data were displayed using GraphPad Prism software. Statistical tests were performed in R and included multivariate permutation test for paired comparison (https://rdrr.io/cran/CNPS/), Shapiro-Wilk normality, paired 2-tailed *t* test, and Wilcoxon signed-rank test (not normally distributed). TCR V-J usage plots were generated using VDJ tools (50). Error bars depicting SEM are displayed when triplicate conditions were performed. Flow cytometry data were analyzed with FlowJo software (Tree Star Inc) or NovoExpress (Agilent).

### Study approval.

Donors recruited via the Welsh Blood Service gave informed consent as part of the donation procedure and samples were used under local ethical approval granted by the School of Medicine Research Ethics Committee (reference 18/56). The patient with AML gave informed consent as overseen by the clinicians at the University Hospital of Wales (UHW), Cardiff under ethical approval number 17/L0/1566231974, granted by the NHS Research Ethics Committee.

### Data availability.

Biological datasets are presented in the Supporting Data Values file. Other data are available from corresponding author upon reasonable request.

## Author contributions

AKS and GD conceived the study. GD, HT, LRT, CR, SD, GAI, LFC, MDC, EB, TM, MEC, DS, LP, MSH, AF, KT, AW, JRH NO, and JF designed research studies, conducted experiments and acquired data. GD, HT, BS and AKS analyzed the data. CA and JZ provided critical reagents/samples. AKS and GD wrote the study.

## Supplementary Material

Supplemental data

Supporting data values

## Version 1 01/02/2025

Electronic publication
