# Supplementary material for: MHC-related protein 1–restricted recognition of cancer via a semi-invariant TCR-α chain
Source: J Clin Invest. 2025 Jan 2;135(1):e181895. doi: 10.1172/JCI181895 (PMC11684821; doi:10.1172/JCI181895)
Supplement: Supplemental data [file jci-135-181895-s065.pdf]

SUPPLEMENTAL MATERIAL

MHC-related protein 1-restricted recognition of cancer via a semi-invariant TCRα chain

Garry Dolton[1*], Hannah Thomas[1*], Li Rong Tan[1], Cristina Rius[1], Stephanie Doetsch[1], Giulia-Andreea Ionescu[1], Lucia F. Cardo[1], Michael D. Crowther[1], Enas Behiry[1], Théo Morin[1], Marine Caillaud[1], Devinder Srai[4], Lucia Parolini[4], Md Samiul Hasan[1], Anna Fuller[1], Katie Topley[1], Aaron Wall[1], Jade R. Hopkins[1], Nader Omidvar[1], Caroline Alvares[2], Joanna Zabkiewicz[2], John Frater[5], Barbara Szomolay[1,3] and Andrew K. Sewell[1,3,6].

[1]Division of Infection and Immunity, Cardiff University School of Medicine, Cardiff, Wales, UK
[2]Division of Cancer and Genetics, Cardiff University School of Medicine, Cardiff, Wales, UK
[3]Systems Immunology Research Institute, Cardiff University Cardiff, Wales, UK
[4]Nuffield Department of Medicine and Department of Chemistry, University of Oxford, Oxford, UK
[5] Nuffield Department of Medicine and NIHR Biomedical Research Centre University of Oxford, Oxford, UK
[6]Division of Infection and Immunity, Kumamoto University, Kumamoto 8600811, Japan

Correspondence to AKS: sewellak@cardiff.ac.uk

This PDF file includes:

- Supplementary Figures S1 to S7
- Supplementary Materials and Methods
- Supplementary references 48-58

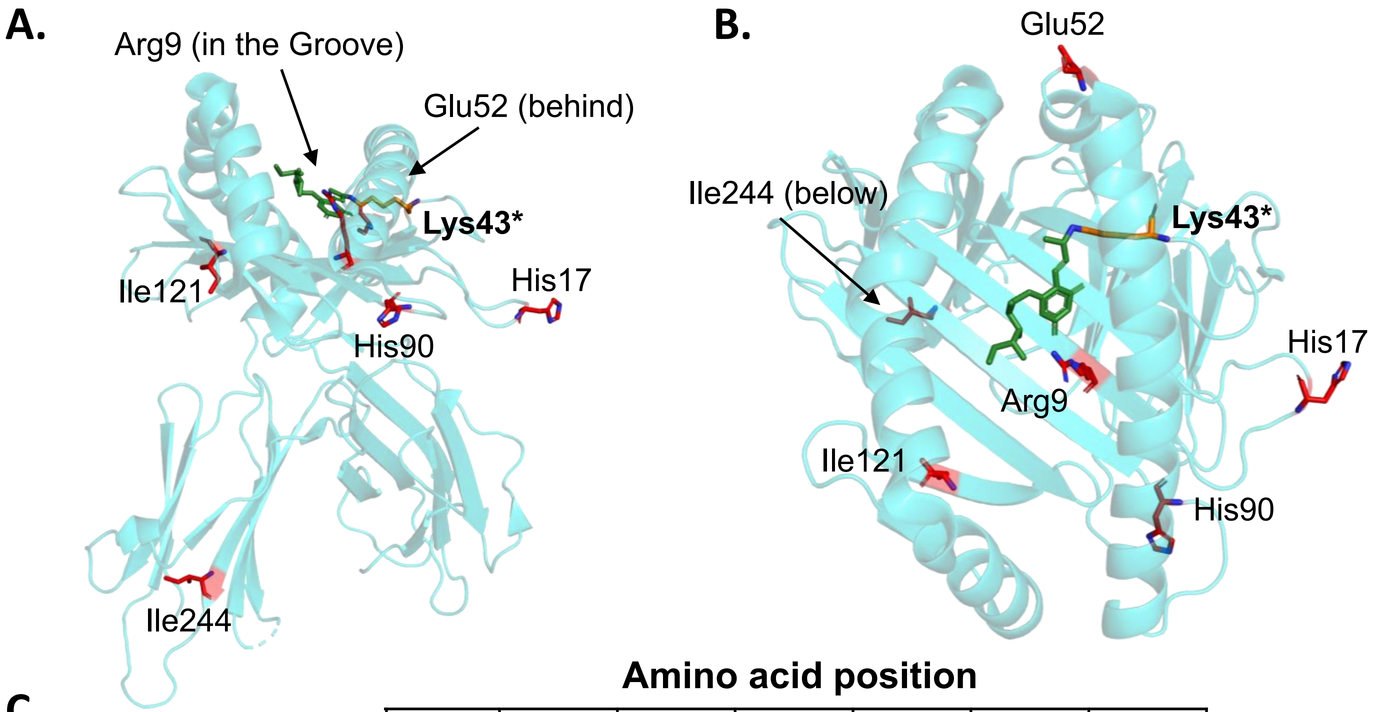

C.

Amino acid position

MR1 allele	9	17	52	90	121	244	304
MR1*01	R	H	E	H	I	I	R
MR1*02	R	**R**	E	H	I	I	R
MR1*03	R	H	E	H	**V**	I	R
MR1*04	**H**	**R**	E	H	I	I	R
MR1*05	R	H	**G**	**Q**	I	**V**	R
MR1*06	R	H	E	H	I	I	**K**

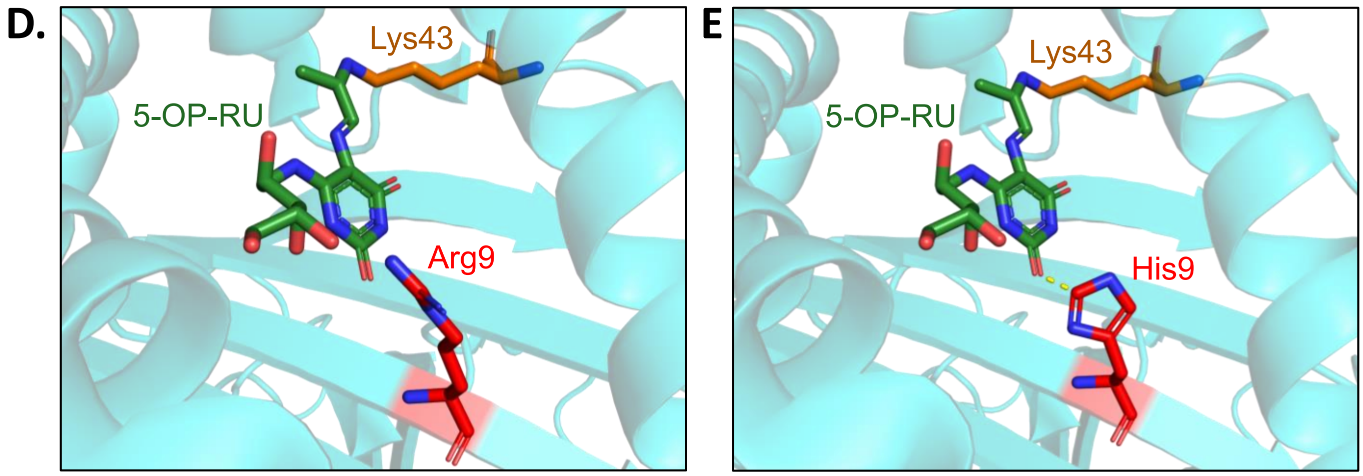

Supplementary Figure 1. 3D structure of MR1 (cyan cartoon) in complex with 5-OP-RU (green sticks) (PDB: 6PUC) from side (**A**) and top down 'TCR view' (**B**) profiles, showing the locations of allelic variation (red sticks) as described by Rozemuller *et al.* (14) (**C**). Lysine 43 (*), responsible for Schiff-base interaction with 5-OP-RU, is highlighted in orange. (**D-E**) close ups of 5-OP-RU including Lys43 and Arg9 (**D**) or predicted position (PyMol) of MR1*04 specific mutation 'His9' (**E**). Yellow dashes in (**E**) highlight Van der Waals force of 1.7 Å.

A.

MR1*01 | MR1*02 H17R | MR1*04 R9H and H17R

B.

Cell line	Tissue	Cancer	Sequencing	MR1		MC.27.759S	MC.7.7G5	K8T-1	K8T-2
C1R	Blood	Lymphoblastoid		*01	*01		*NI*		
MM909.24	Skin	Melanoma		*01	*04	T-cell lines of this study			
A549	Lung	Carcinoma		*01	*04		*NI*		
K-562	Bone marrow	CML		*01	*01		*NI*		
MCF-7	Breast	Adenocarcinoma		*01	*02		*NI*		
ACHN	Kidney	Adenocarcinoma		*01	*01				
MIA PaCa-2	Pancreas	Carcinoma		*01	*01				
THP-1	Blood	AML		*01	*01	ME216 T-cell line			
FM3	Skin	Melanoma		*01	*01				
BxPC-3	Pancreas	Carcinoma		*01	*01				
FM86	Skin	Melanoma		*01	*01				
SiHa	Cervix	Carcinoma		*01	*02		*NI*		
MEL624	Skin	Melanoma		*01	*02		*NI*		
FM72	Skin	Melanoma		*01	*01				
MEL526	Skin	Melanoma		*01	*01				
FM74	Skin	Melanoma		*01	*01				
Kasumi-3	Blood	AML		*01	*02				
MOLT-3	Blood	ALL		*01	*01				
MDA-MB-231	Breast	Adenocarcinoma		*01	*01		*NI*		
FM88	Skin	Melanoma		*01	*02				
ES-5838	Bone	Ewing's Sarcoma		*01	*01				
RD-ES	Bone	Ewing's Sarcoma		*01	*01				
6647	Bone	Ewing's Sarcoma		*01	*01				
TC-71	Bone	Ewing's Sarcoma		*01	*01				
NCI-LG	Bone	Ewing's Sarcoma		*01	*01				
A2780	Ovarian	Adenocarcinoma		*01	*01		*NI*		

C.

U266	Blood	*01	*01	
COLO205	Colon	*01	*01	
U2OS	Bone	*01	*02	
RCC17	Kidney	*01	*01	
Jurkat	Blood	*01	*01	
LnCAP	Prostate	*01	*02	
FM45	Melanoma	*01	*01	

In-house sequencing

Crown Biosciences database

Supplementary Figure 2. MR1 alleles expressed by the cancer cells used in this study. Established by in-house sequencing and/or database searching. (**A**) MR1 alleles identified for this study and highlight color used for each throughout the figure. (**B**) Cancer cells used in the study with their MR1 alleles indicated. Cancer cells tested with MC.27.759S (759S), MC.7.G5 (7G5), K8T-1 and K8T-2 TCRs indicated by the grey shade boxes. *NI* indicates testing also performed in *Crowther et al* study (17). (**C**) Other cancer cell lines tested with MC.7.G5 clone in *Crowther et al* study (17).

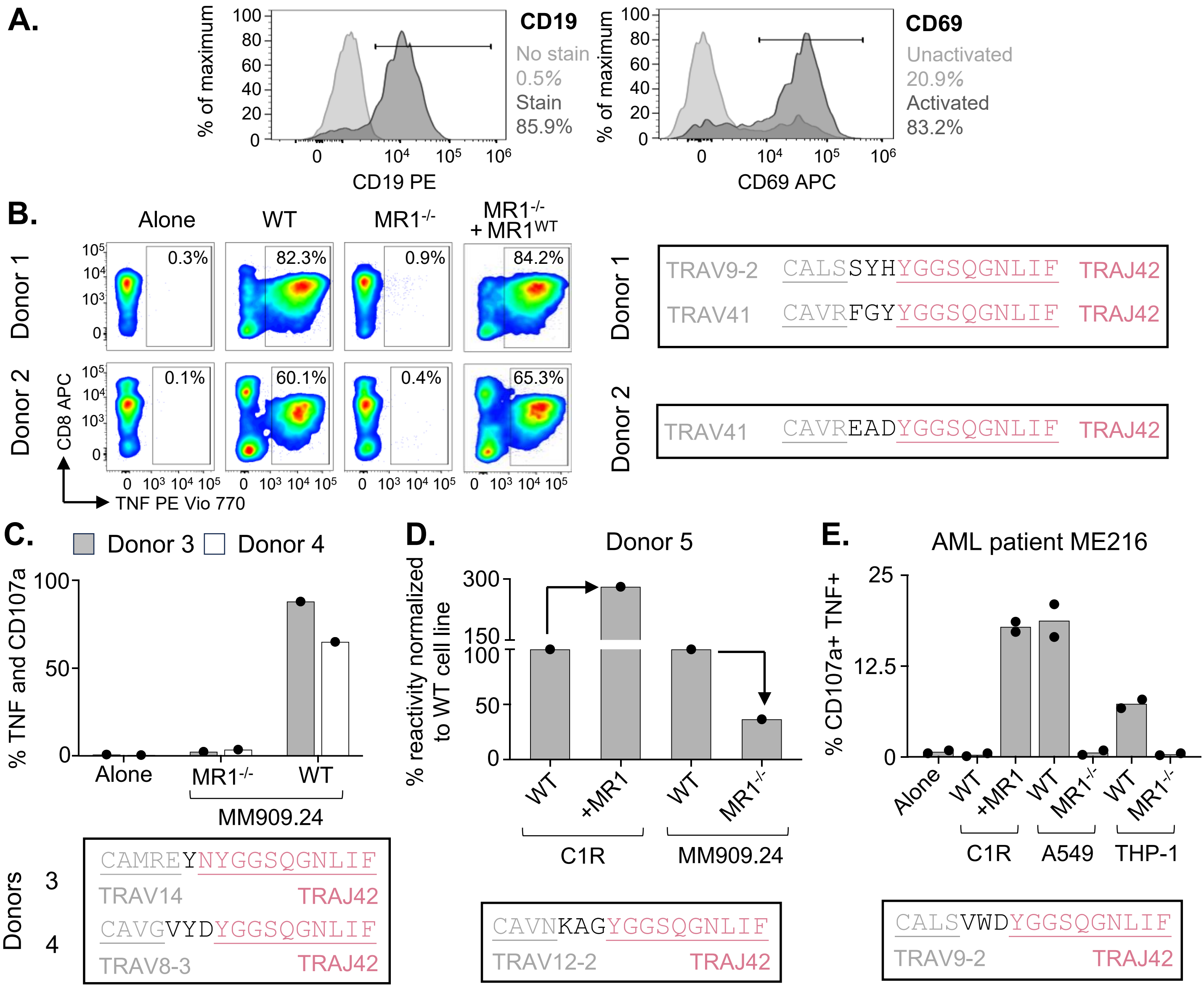

Supplementary Figure 3. Sequencing of cancer-activated MR1-restricted TCRs from multiple donors reveals a *TRAJ42* bias. (A) Unactivated and activated (TLR9 ligand) purified (CD19^{+}) healthy B-cells used as target cells for MC.27.75S and JMA TCR transduced Jurkat cell. B-cell expressed CD69 used as a marker of successful activation. (**B**) Left: T107 assay of T-cell lines from donors 1 and 2 tested against MM909.24 melanoma: WT, MR1 knockout ($^{-/-}$) and MR1$^{-/-}$ + MR1*01. Right: MR1-reactive *TRAJ42* containing TCRα chains from the T-cell lines. Germline amino acid residues of variable (V) and joining (J) regions are underlined and inserted amino acids in black and not underlined. (**C**) T-cell lines from donors 3 and 4 tested in a T107 assay (4h) against WT and engineered melanoma MM909.24 cells. MR1-reactive CDR3α from these lines that use *TRAJ42* are shown below. (**D**) T-cell line from Donor 5 tested against C1R WT and C1R+MR1*01 and melanoma MM909.24 +/- MR1. Data has been normalized to reactivity seen for C1R WT or MM909.24 WT melanoma cells. C1R + MR1*01 and MM909.24 WT reactive CDR3α using *TRAJ42* are shown below. These TCRs did not appear in T-cells reacting towards WT C1R cells and MR1$^{-/-}$ MM909.24 melanoma cells. (**D**) T-cell line from AML patient ME216 tested against C1R WT and C1R + MR1*01, lung cancer A549 WT and overexpressing scβ2M-MR1*01, and AML THP-1 WT and overexpressing MR1*01. T107 assay for 4h. MR1 reactive CDR3α using *TRAJ42* is shown below.

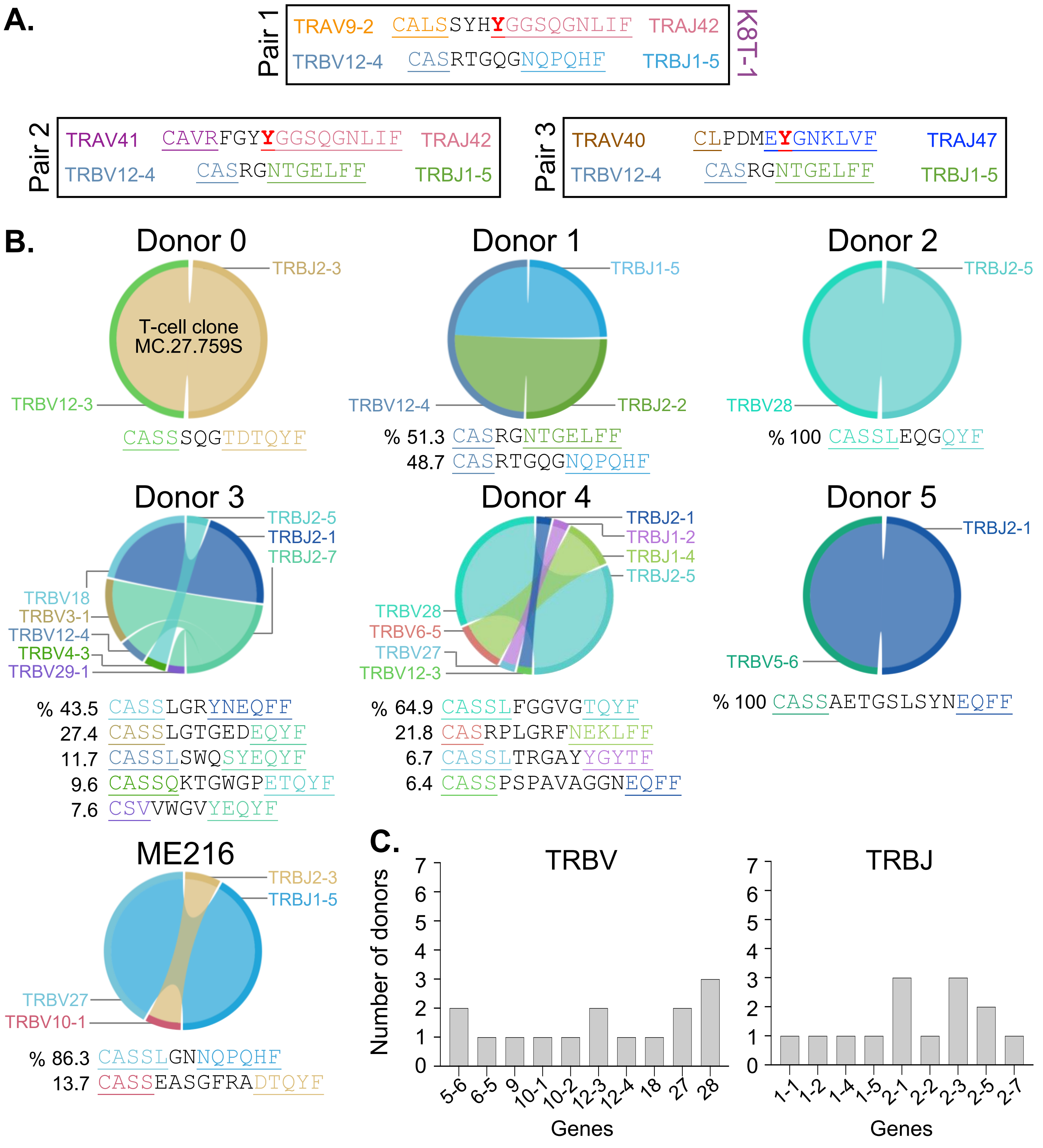

Supplementary Figure 4. *TRBV/TRBJ* usage of cancer-activated MR1-restricted T-cells. (A) Paired TCRs from donor 1. (**B**) *TRBV* and *TRBJ* gene usage of MR1-reactive T-cells from six healthy donors (clone MC.27.759S and T-cells lines) and one AML patient (ME216). Circos plot show *TRBV* (V) genes on the right and *TRBJ* (J) genes on the left, with the size of the outer arc corresponding to the relative frequency of the TRAV or TRAJ genes. The ribbons between the arcs represent TRAV-TRAJ pairings. (**C**) Summary of TRBV (left) and TRBJ (right) genes used by MR1 reactive T-cells from six healthy and one AML patient.

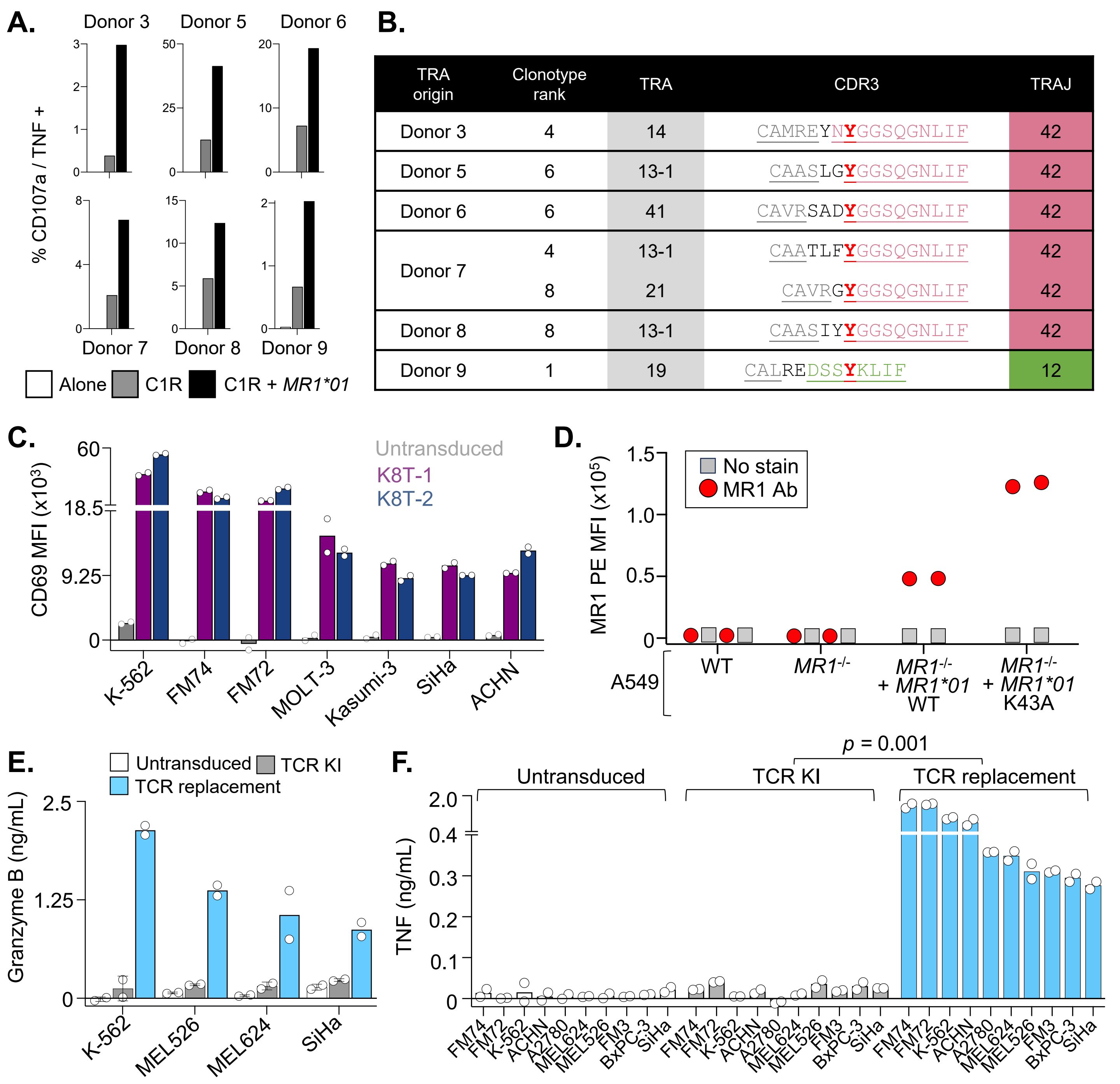

Supplementary Figure 5. *TRAJ42* usage and functional testing of cancer-activated MR1-resticted T-cells. (**A**) Eight healthy donors were primed with C1R cells overexpressing *MR1*01*. We aimed to establish if *TRAJ42* containing TCRs could be seen after one priming with MR1 expressing cells, without the function-based enrichment(s) detailed in **Figure 1**. After 2 weeks, reactivity was assessed towards C1R and C1R + *MR1*01* cells by T107 assay. Six (data shown) of the seven donors showed MR1 dependent reactivity. Donors 3 and 5 had already been used for the study and were included as positive controls. (**B**) T-cells from the lines in (A) were sorted by flow cytometry based on TNF/CD107a upregulation towards C1R + *MR1*01* cells. TCR sequences with *TRAJ42* containing TCRα chains appearing in 5/6 donors. CDR3α chains for the *TRAJ42* TCRs are shown for five donors, also for the donor with a dominant *TRAJ12* gene usage, which is also used by MAIT cells. (**C**) CD69 assay (24h) of K8T-1 and K8T-2 TCR transduced Jurkat cells with *MR1*01* cancer lines: Duplicate conditions. (**D**) MR1 staining of A549 cancer cells: WT, MR1 knockout ($MR1^{-/-}$) by CRISPR-Cas9, and $MR1^{-/-}$ with transgenic expression of scβ2M-MR1*01 WT or scβ2M-MR1*01 K43A. Duplicate staining conditions. (**E**) Donor 216T CD8 T-cells, either untransduced, MC.7.G5 TCR KI or MC.7.G5 TCR replaced in an overnight activation assay with cancer cells, followed by Granzyme B ELISA. Duplicate conditions. (**F**) Donor 216T CD8 T-cells, either untransduced, MC.7.G5 TCR KI or MC.7.G5 TCR replaced in an overnight activation assay with cancer cells, followed by TNF ELISA. P value for a multivariate permutation test for paired comparison. **Cancer cells:** leukemia (K-562, MOLT-3, Kasumi-3), melanoma (FM74, FM72, FM3, MEL625, MEL526), cervical (SiHa), ovarian (A2780), pancreatic (BXPC-3) and kidney (ACHN).

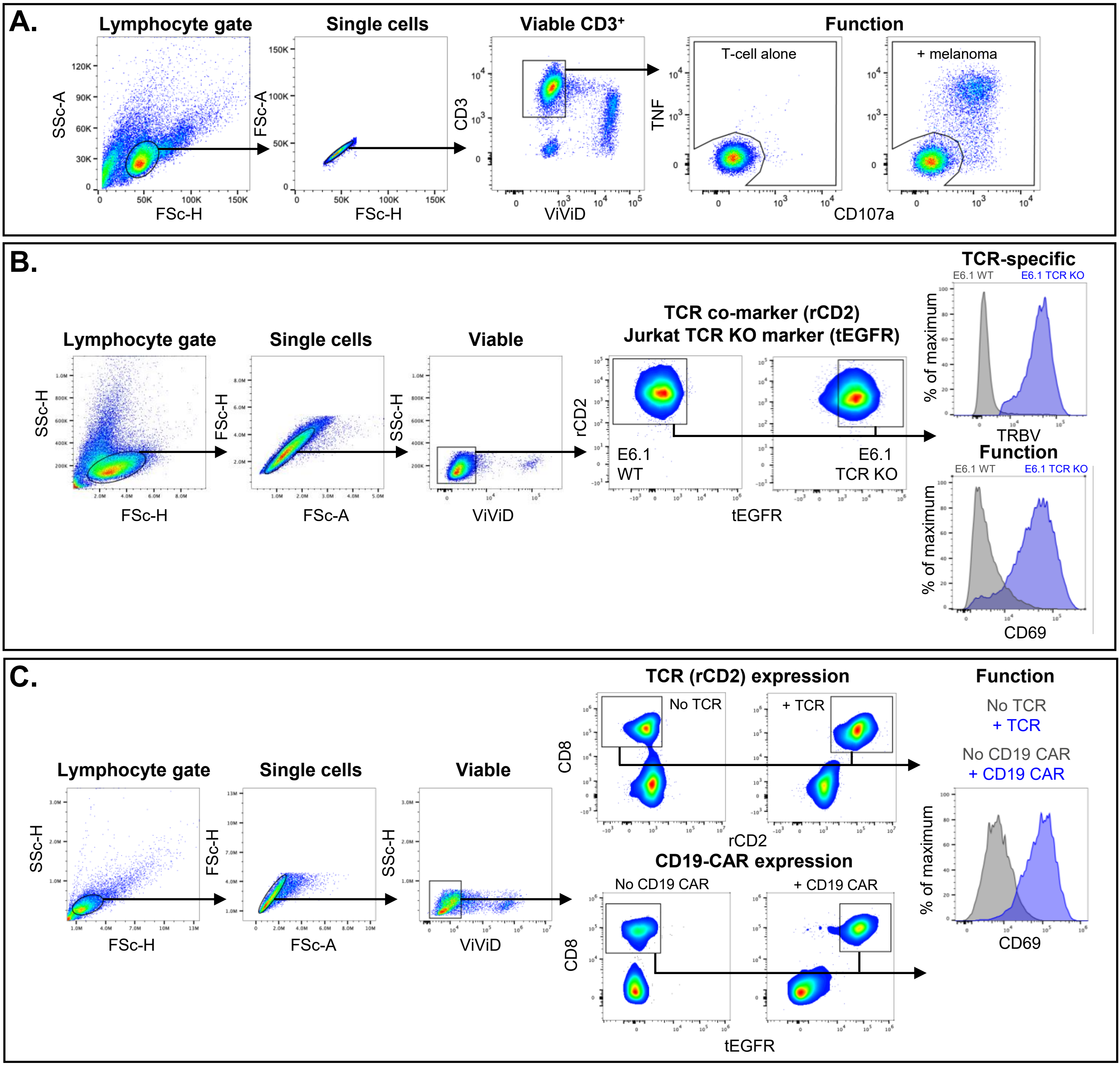

Supplementary Figure 6. Flow cytometry Gating strategies. (**A**) Intracellular cytokine staining. Functional gating set on T-cells alone. (**B**) TCR expression and reactivity of MR1 restricted TCRs (rCD2 as a co-marker) in E6.1 Jurkat cells, either wildtype or TCR knockout (tEGFR as knockout co-marker). (**C**) Triple Parameter Reporter (TPR) Jurkat cell CD69 assay. Rat (r) CD2 used as a co-marker for TCR expression and tEGFR as a co-marker for CD19-CAR expression.

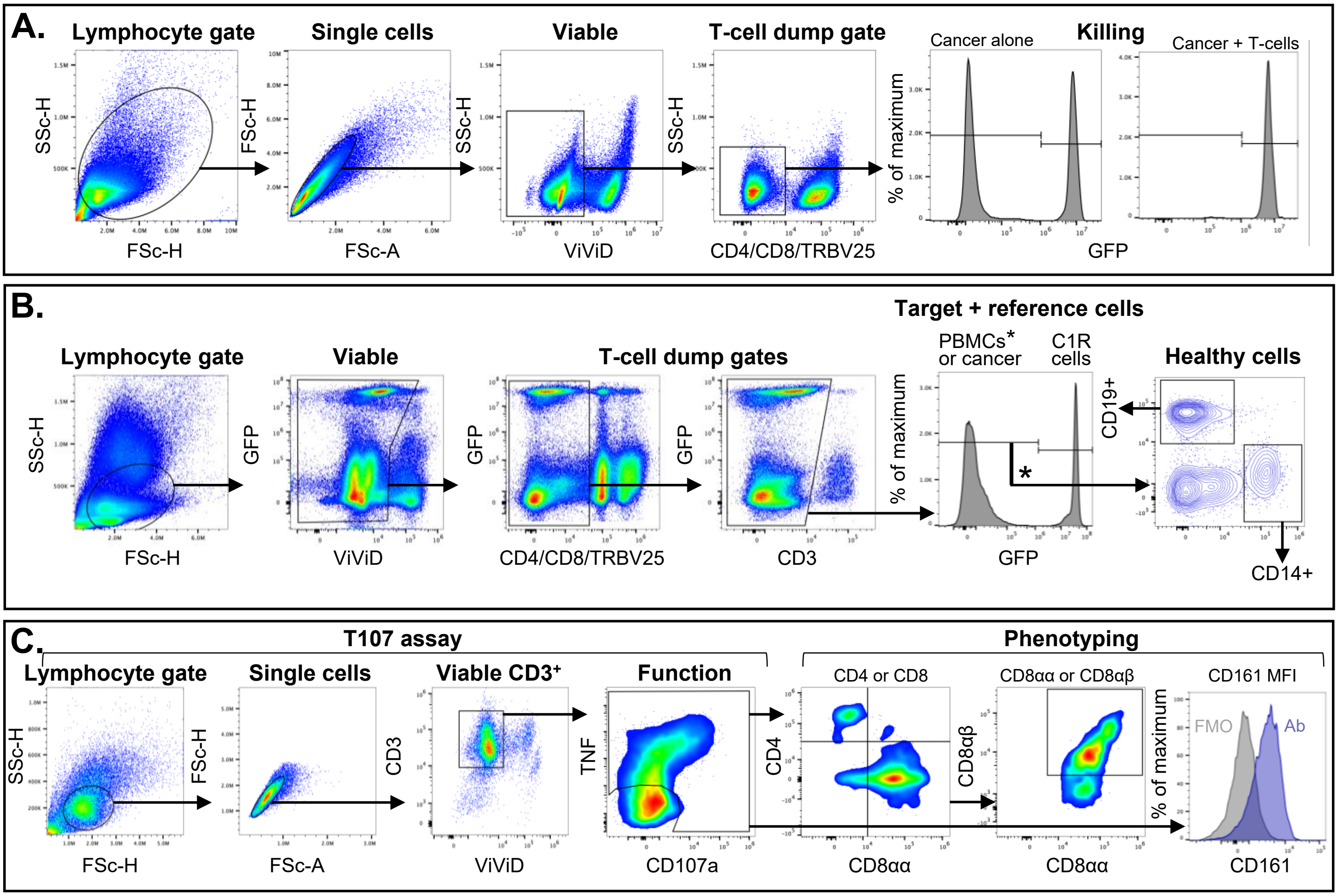

Supplementary Figure 7. Flow cytometry Gating strategies. (**A**) T-cell clone killing assay with cancer cells. GFP⁺ C1R cells added to assay wells before harvest and staining to enable % killing to be calculated. (**B**) Killing assays with TCR-T cells versus cancer cells or healthy cells. GFP⁺ C1R reference cells added immediately prior to harvest and staining for flow cytometry. Cell number events of GFP⁺, CD19⁺ and CD14⁺ cells used to assess killing of healthy B-cell and monocyte subsets. Percentage of cancer cells and GFP⁺ cells used to calculate killing of cancer cells. (**C**) T107 assay in combination with phenotyping for CD8 chains and CD161 levels. Functional gating set on T-cell alone and/or T-cells with MR1 knockout cancer cells. Phenotyping gating set on controls without antibodies.

Supplementary Materials and Methods

Healthy donors

Blood samples from healthy donors were sourced from the Welsh Blood Service (Velindre NHS Trust, Wales, UK) as EDTA treated 'buffy coats' and ethical approval granted by the School of Medicine Research Ethics Committee (reference 18/56). Each buffy coat was seronegative for HIV-1, HBV and HCV. Blood and cells derived thereof were handled in accordance with Cardiff University guidelines in alignment with the United Kingdom Human Culture Act 2004. Blood from the WBS was diluted 1:1 with R10 (RPMI-1640 supplemented with 10% heat-inactivated FBS, 100 U/ml penicillin, 100 µg/mL streptomycin and 2 mM L-glutamine (all Merck)) and placed on a Cole-Parmer™ Stuart™ roller-mixer overnight at 9-11 rpm and room temperature. The following morning the blood was further diluted 2:1 (blood:RPMI-1640) then PBMCs separated using conventional density gradient centrifugation with Sigma-Aldrich Histopaque 1077 (Merck). Red blood cells were removed using lysis buffer (155 mM NH_4Cl, 10 mM $KHCO_3$ and 0.1 mM EDTA, pH7.2-7.4) for 10 min at 37°C.

Acute myeloid leukemia patient

A blood sample from acute myeloid leukemia (AML) patient ME216 (male) was provided through a collaboration with clinicians at the University Hospital of Wales (UHW), Cardiff. Ethical approval for the sample was granted and consent taken under the ethical approval number 17/L0/1566231974, granted by the NHS Research Ethics Committee. Venous blood was processed as above for the isolation of PBMCs.

Cancer cell lines

Cell lines were regularly tested for mycoplasma using a MycoAlert® mycoplasma detection kit according to the manufacturer's instructions (Lonza, Basel, Switzerland) and cultured at 37°C with 5% CO2 in R10 (as above), D10 or D10-F12 (as for R10 but with DMEM or DMEM-F12 respectively). Adherent cell lines were passaged when 50-80% confluent by detachment from tissue culture plates or flasks with D-PBS and 2 mM EDTA, then splitting 1:3-1:20. Suspension cell lines were passaged at least once a week by 1:5-1:50 dilution. Cutaneous melanoma lines were provided by the CCIT-DK (FM) or sourced locally (culture medium in brackets): FM3 (R10), FM86 (R10), FM72 (D10-F12), FM74 (D10-F12), FM88 (R10), MEL624 (R10), and MEL526 (R10). Melanoma line from patient MM909.24 (R10) were generated from metastatic lesions at the CCIT-DK (48). Adherent cancer cell line sourced locally: lung carcinoma A549 (R10), breast adenocarcinomas MCF-7 and MDA-MB-231 (R10), cervical carcinoma SiHa (D10-F12), pancreatic carcinoma MIA PaCa-2 (D10-F12) and ovarian adenocarcinoma A2780 (D10). Suspension cell lines sourced locally: B-lymphoblastoid C1R (R10), acute myeloid leukemia THP-1 (R10), acute lymphoblastic leukemia MOLT-3 (R10) and chronic myeloid leukemia K-562 (R10). Kidney cancer cell line ACHN from Cell

Line Service (Baden-Wuerttember, Germany) were cultured in D10 as adherent cells. Acute myeloid leukemia line Kasumi-3 was purchased from DSMZ (Braunschweig, Germany) and cultured as suspension cells in R10. Pancreatic carcinoma BxPC-3 were from DSMZ (Braunschweig, Germany) and cultured in R10 as adherent cells. Ewing's Sarcoma cell lines ES-5838, RD-ES, 6647 and TC-71 were kindly provided by Robbie Majzner, Stanford University and cultured as adherent cells in R10 media with 10 mM HEPES. Jurkat TCR KO CD8αβ+ triple parameter reporter (TPR) cells (49) were provided by Professor Peter Steinberger (Vienna, Austria). E6.1 Jurkat cells were purchased from the ATCC. Jurkat cells were cultured in R10 as suspension cells and passaged 1:2-1:20 1-3 times a week.

Sequencing of MR1

Cell line MR1 allotype data were downloaded from the Crown Bioscience XenoBase® database (https://xenobase.crownbio.com/) in January 2024 (freely available with registration). For in-house sequencing and MR1 allomorph identification, genomic DNA was purified from 2-5 $x10^6$ cells using a PureLink genomic DNA mini kit (ThermoFisher Scientific). Primers for PCR: forward 5'-CACACGTGCACACACAGAGGTG and reverse 5'-GGACAGTCCAGAAGATGCACAGG. PCR mix using Phusion High Fidelity PCR Master Mix (ThermoFisher Scientific): 1 µL forward primer (from 100 µM stock), 1 µL reverse primer (from 100 µM stock), 25 µL 2x Phusion master ix, 4 µL (200 ng) of DNA template and 19 µL of DNAse-free H_2O. PCR cycles: 1X denaturation at 98°C for 3 min, 35X denaturation 98°C for 10 sec, annealing 67°C for 15 sec, extension 72°C for 30 sec, then finally 1X extension at 72°C for 5 min. PCR product run on a 1% agarose gel at 120V for 1 h, and the 904bp band cut and cleaned with Monarch DNA gel extraction kit (New England BioLabs, Ipswich, MA, USA). Sequencing primers: forward 5'- GAGCTCTTACGTCCTGTCCAGG and reverse 5'-GGACAGTCCAGAAGATGCACAGG. Sequencing data was analyzed using SnapGene software.

MR1 knockout and overexpression

C1R cells overexpressing MR1*01 were generated as previously described (18) and maintained as lines expressing >95% MR1 based on antibody staining with clone 26.5 (BioLegend). A549 MR1 knockout clone 9 was generated as previously described (50), and the same methodology used to create MR1 knockout melanoma MM909.24 (clone 4) and THP-1 (clone 5) cell lines. MM909.24 clone 4 overexpressing MR1*01 was created as above for C1R cells using a codon optimized MR1 heavy chain. A549 MR1 knockout clone 9 cells with re-expressed wildtype MR1*01 or K43A MR1*01 were created as a single chain (sc) construct with β-2-microglobulin (*B2M*). A codon-optimized gene for *MR1* without its signal sequence (uniport.org, Nov. 2022) was fused to *B2M* via a GGGGSGGGGSGGGGS linker, which was synthesized by GeneArt (Life Technologies). A strep-II tag2 was added to the *scB2M/MR1* construct by site directed mutagenesis using the following

oligonucleotides: Strep-II forward oligo (5' CCGACAGATGGTCTCACCCCCAATTTGAAAAACTCGAGAGCGGCTCCGGCGAG 3') and Strep-II reverse oligo (5' CTCTCGAGTTTTTCAAATTGGGGGTGAGACCATCTGTCGGGGGTGGGCAGG 3'). A K43A substitution was then added by site directed mutagenesis using the following oligonucleotides: K43A MR1 forward (5' GCCGAGCCCAGAGCCCCCTGG 3') and K43A MR1 reverse (5' GGCCTGTCTGGTCACGCTGTCG 3'). Oligonucleotides were purchased via the Eurofins Genomics Website (Luxembourg, EU). The *scβ2M/MR1* strep-II construct was inserted into the pELNS backbone with rCD2 co-marker: XbaI-Kozak-*B2M*-GS linker-*MR1*-XhoI-*P2A*-*rCD2*-SalI-Stop. Confirmation of MR1 transduction was carried out 7 days later by staining with MR1 antibody (clone 26.5, BioLegend) and anti-rCD2 antibody (clone OX34, BioLegend). Cell lines were maintained at >95% MR1$^+$ based on staining with antibody clone 26.5 and achieved by flow cytometry sorting using a BD FACS Aria III (BD Biosciences), performed by Central Biotechnology Services at Cardiff University.

Priming and enrichment of cancer reactive T-cells

PBMCs (8 x10^6 per well of a 24 well plate) from healthy donors were co-cultured with irradiated (3100 Gy) C1R cells (0.8 x10^6) overexpressing MR1*01, in 2 mL of priming media (RPMI-1640 supplemented with 10% heat-inactivated FBS, 100 U/ml penicillin, 100 μg/mL streptomycin, 2 mM L-glutamine, 20 IU/mL IL-2 (Proleukin®; Prometheus, San Diego, CA), 1X non-essential amino acids solution, 1 mM sodium pyruvate and 10 mM HEPES buffer (all from Merck KGaA, St Louis, Missouri, USA, unless stated otherwise). As sample size was limited from AML patient ME216 (approximately 10 mL of blood), CD8 T-cells were purified using anti-CD8 microbeads according manufacturer's instructions (Miltenyi Biotec) then amplified with CD3/CD28 Dynabeads (Life Technologies) at 1:1 bead:T-cell ratio in T-cell media (R10 with 2 mM L-glutamine, 25 ng/mL IL-15 (Miltenyi Biotec, Bergisch Gladbach, Germany), 200 IU/mL IL-2 (Proleukin®; Prometheus, San Diego, CA), 1X non-essential amino acids solution, 1 mM sodium pyruvate and 10 mM HEPES buffer (all from Merck KGaA, St Louis, Missouri, USA, unless stated otherwise)). After 3 weeks, the amplified T-cells (3 x10^6 per well of a 24 well plate) from ME216 were co-incubated with irradiated (3100 Gy) C1R cells (0.8 x10^6) overexpressing MR1*01, in 2 mL of priming media. Two weeks following priming, cells were isolated using a magnetic TNF enrichment kit according to the manufacturer's instructions (Miltenyi Biotec) following incubation with MM909.24 melanoma cell lines, either WT or MR1 knockout overexpressing MR1*01. Enriched cells were incubated overnight in a well of a 96U well plate, then expanded using up to 0.1 x10^6 T-cells per well of a 96U well plate, with 5 x10^4 irradiated (3100 Gy) PBMCs from three allogenic donors provided by the Welsh Blood Service, and 1 μg/mL of L-phytohemagglutinin (PHA) (Remel, ThermoFisher Scientific) in 0.2 mL of T-cell media with 20 IU/mL of IL-2 and 25 ng/mL of IL-15. After 2-3 weeks of expansion the T-cells were tested for reactivity.

Culture media was changed thrice weekly, and T-cells monitored for expansion and if needed split over multiple wells. For some of the T-cell lines the TNF enrichment step was repeated.

TCR sequencing

Flow cytometric sorted populations of T-cells were placed in RNAprotect Cell Reagent (Qiagen). TCR sequencing was performed as previously described (51). RNA extraction was carried out using the RNEasy Micro kit (Qiagen). cDNA was synthesized using the 5'/3' SMARTer kit according to the manufacturer's instructions (Takara Bio, Paris, France). The SMARTer approach used a Murine Moloney Leukaemia Virus (MMLV) reverse transcriptase, a 3' oligo-dT primer and a 5' oligonucleotide to generate cDNA templates flanked by a known, universal anchor sequence at the 5'. A Step-Out PCR was performed using a pair of primers consisting of 3' TRAC or TRBC-specific reverse primer (Eurofins Genomics) and a 5' universal anchor-specific forward primer (Takara Bio, Paris, France). All samples were used for the following PCR reaction: 2.5 μL template cDNA, 0. 5 μL High Fidelity Phusion Taq polymerase, 10 μL 5X Phusion buffer, 0.5 μL DMSO, 1 μL dNTP Mix (stock concentration of 10 mM of each) (all from ThermoFisher Scientific, UK), 1 μL of TRAC or TRBC-specific primer (10 μM stock), 5 μL of 10X anchor-specific universal primer (Takara Bio, Paris, France), and nuclease-free water for a final reaction volume of 50 μL (cycling conditions: 5 min at 94°C, 30 cycles of 30 s at 94°C, 30 s at 63°C for alpha chains or 30 s at 66°C for beta chains, 120 s at 72°C). Subsequently, 5 μL of the Step-out PCR products were taken for a nested PCR, using 1 μL of barcoded forward (universal) and reverse (*TRAC* or *TRBC*) primers (10 μM stock) (Eurofins Genomics), 0.5 μL High Fidelity Phusion Taq polymerase, 10 μL 5X Phusion buffer, 0.5 μL DMSO, 1 μL dNTP Mix (stock concentration of 10 mM each) and nuclease-free water for a final reaction volume of 50 μL (cycling conditions: 5 min at 94°C, 30 cycles of 30 s at 94°C, 30 s at 62°C, 120 s at 72°C, and a final 10 min at 72°C). The final PCR products were loaded on a 1% agarose gel and purified with the Monarch® gel extraction kit (New England Biolabs). Purified products were sequenced on an Illumina MiSeq instrument using the MiSeq v2 reagent kit (Illumina, Cambridge, UK) according to the manufacturer's instructions. Sequence analysis was performed using MiXCR software (v3.0.7) (52). Public TCR clonotypes were identified using the VDJdb database (53). TCRs were excluded from display below 5% (main figures) or 2% (supplementary figures) of sequencing reads, with the percentages displayed being re-calculated post this refinement.

Paired chain single-cell TCR sequencing

Paired alpha and beta chain V(D)J sequences were obtained using a novel single cell encapsulation methodology. Single cell encapsulation was achieved using a flow-focusing microfluidic device (fabricated via Polydimethylsiloxane (PDMS) soft lithography using Sylgard 184 silicone elastomer kit (Dow Corning) with a 10:1 ratio of base to curing agent). The device was designed with two inlets for the dispersed phase. 008-FluoroSurfactant (Ran Biotechnologies) in 3M Novec 7500 Engineered Fluid was the

continuous phase. Cells were re-suspended prior to lysis. The first emulsification step was collected on ice before being broken using 1H,1H,2H,2H-Perfluoro-1-octanol (Merk). Thermocycling conditions were as reported elsewhere (54)

MC.7.G5, MC.27.759S and T-cell line maintenance

T-cell clones MC.7.G5 (18) and MC.27.759S, and cancer-reactive T-cell lines were cultured in T-cell media (RPMI-1640 supplemented with 10% heat-inactivated FBS, 100 U/ml penicillin, 100 µg/mL streptomycin, 2 mM L-glutamine, 25 ng/ml interleukin IL-15 (Miltenyi Biotec, Bergisch Gladbach, Germany), 200 IU/mL IL-2 (Proleukin®; Prometheus, San Diego, CA), 1X non-essential amino acids solution, 1 mM sodium pyruvate and 10 mM HEPES buffer (all from Merck KGaA, St Louis, Missouri, USA, unless stated otherwise). Every 2-3 weeks, 0.2 x10^6 of MC.7.G5, MC.27.759S or T-cell were expanded per well of a 24 well plate, using 4 x10^6 irradiated (3100 Gy) PBMCs from three allogenic donors provided by the Welsh Blood Service and 1 µg/mL of L-phytohemagglutinin (PHA) (Remel, ThermoFisher Scientific) in 2 mL T-cell media with 20 IU/mL of IL-2. MC.7.G5 and MC.27.759S were used for functional assays from day 14 onwards post expansion.

Healthy cells

Monocytes and B-cells were purified from freshly isolated PBMCs procured from the Welsh Blood Service using anti-CD14 or anti-CD19 microbeads respectively according to the manufacturer's instructions (Miltenyi Biotec). Purified cells were used immediately for assays. Alternatively, whole PBMCs were added to assay wells for flow cytometry based killing assays, then CD14 and CD19 antibodies used to identify the different cell subsets (see below for more details). Healthy B-cells were activated 24h prior to assay with 2 µM of TLR9 ligand ODN 2006 (Miltenyi Biotec) and activation confirmed using CD69 APC conjugated antibody (clone FN50, BioLegend).

***Mycobacterium smegmatis* infection**

The phagocytic A549 cell line was loaded with *Mycobacterium smegmatis* (strain mc2155, kindly provided by Professor Matthias Eberl, Cardiff University, available at ATCC catalogue 700084). For culture, glycerol stock derived bacterium were inoculated into Lemco media (5 g/L Lemco powder, 10 g/L Tryptone, 5 g/L NaCl (all from Fisher Scientific) and 0.25% Tween-80 (Merck)) and grown for 72 h at 37 °C and 170rpm. Prior to infection of A549 cells, *M. smegmatis* cultures were harvested and centrifuged at 2740 x *g* for 20 min, then washed with excess antibiotic free R10 at 2740 x *g* for 20 min. In preparation for infection, 3.5 x10^6 A549 cells (WT and MR1 knockout) were cultured for 3 days in a T175 flask in R10, then media removed and replaced with antibiotic-free R10 overnight. On the morning of infection the media was removed from the A549 cultures and replaced with fresh antibiotic free R10 (20 mL), then *M. smegmatis* added to A549 cells at a multiplicity of

infection of 100-300:1 (bacteria to A549 cells) based on retrospective colony forming units (CFU) from Lemco agar plates (5 g/L Lemco powder, 10 g/L Tryptone, 5 g/L NaCl and 15 g/L agar, Fisher Scientific) for 2 h in antibiotic-free R10, followed by 2h incubation with R10 containing 100 U/mL penicillin and 100 µg/mL streptomycin (Life Technologies). A549 cells were washed to remove extracellular *M. smegmatis*. Control uninfected cells (A549 WT and MR1 knockout) were mock treated as if they had been co-incubated with bacteria.

Intracellular cytokine staining assay

Cells were washed from culture medium and incubated in R5 (as for R10, but with 5% FBS) for 24 h prior to activation. Subsequently, cells were incubated at 37°C for 4 h, with and without target cells, at a 1:1 ratio, in 0.2 mL of R5 per well of a 96U well plates with GolgiStop and GolgiPlug (both from BD Biosciences), according to the manufacturer's instructions. Cells were stained with viable LIVE/DEAD™ Fixable Violet Dead (ViVid) Cell Stain Kit (ThermoFisher Scientific), for 5 min at RT, then without washing stained CD3 PerCP conjugated (clone UCHT1, BioLegend) and CD8 APC Vio770 (clone BW135/80, Miltenyi Biotec) antibodies for 20 min on ice. Cells were prepared for intracellular cytokine staining (ICS) by incubation with Cytofix/Cytoperm (BD Biosciences) according to the manufacturer's instructions (including wash steps), before staining for 20 min on ice with TNF PE Vio770 (clone cA2, Miltenyi Biotec) and CD107a FITC (clone H4A3, BD Biosciences) antibodies and data acquired on a BD FACS Canto II (BD Biosciences). Gating strategies shown in **Supplementary Figure 6A**.

Lentivirus plasmids and transfection

For transgene expression the Snap Fast plasmid (pSF) (Oxgene, Oxford Genetics Ltd, Littlemore, Oxford, UK) was used. The pSF plasmid was custom-modified by removal of a XhoI site present in the plasmid backbone and a stop codon inserted after the SalI site. Alternatively, the pELNS expression plasmid was used, which was generously provided by Dr. James Riley (University of Pennsylvania, PA, USA). For the knockout of genes the pLentiCRISPR v2 plasmid (Addgene plasmid # 52961) was used and a kind gift from Feng Zhang (55). Each of these plasmids was used in conjunction with envelope plasmid pMD2.G (gift from Didier Trono, Addgene plasmid # 12259), and packaging plasmids pMDLg/pRRE (gift from Didier Trono, Addgene plasmid # 12251) and pRSV-REV (gift from Didier Trono, Addgene plasmid # 12253). Lentivirus intended for Jurkat and cancer cells was generated in 6-well plates: Day 0, 1.25 x10^6 HEK293T cells plated in 3 mL of D10. Day 1, transfection with plasmid mix (pSF/pELNS/pLentiCRISPR v2: 1.52 µg, pMD2.G: 0.72 µg, pMDLg/pRRE: 1.83 µg pRSV-REV: 1.83 µg) at a 1:3 µg ratio with Polyethylenimine (PEI) (Merck Group) and made up to 300 µL with Opti-MEM (ThermoFisher Scientific). The plasmid/PEI amalgamations were allowed to incubate at RT for 15 min, after which they were added dropwise to the HEK293T cells. Day 2: media was changed for 3 mL of D10. Day 3: supernatant harvested and stored at 4°C.

Day 4: supernatant harvested and combined with the day 3 harvest, centrifuged at 800 x g for 5 min then filtered through a 0.4 µm filter. Lentiviral supernatants were not concentrated and either directly employed for transduction or preserved at -80°C, being defrosted (on ice) only once prior to transduction. To generate lentivirus for primary CD8 T-cells the process was scaled-up to T175 flask: Day 0, 5-6 $x10^6$ cultured in T175 flaks in 20 mL of D10. Day 1, transfection with plasmid mix (pSF/pELNS/pLentiCRISPR v2: 30 µg, pMD2.G: 7.5 µg, pMDLg/pRRE: 15 µg pRSV-REV: 15 µg) at a 1:3 µg ratio with PEIpro (VWR, Pennsylvania, USA) and made up to 750 µL with Opti-MEM. The plasmid/PEIpro was allowed to incubate at RT for 15 min, after which they were added dropwise to the HEK293T cells. Day 2: media was changed for 20 mL of D10. Day 3: supernatant harvested and stored at 4°C. Day 4: supernatant harvested and combined with the day 3 harvest, centrifuged at 800 x *g* for 5 min and filtered through a 0.4 µm filter, then concentrated using an Optima XPN-80 Ultracentrifuge (Beckman Coulter, Brea, CA) and SW28 rotor, at 4°C and 141,000 x *g* for 2 h. Supernatants were discarded and pellets resuspended in 200 µL of T-cell medium containing 200IU/mL of IL-2 and 25 ng/mL of IL-15 and used fresh for transduction without freezing.

TCR transduction of Jurkat cells

For TPR Jurkat cells expressing CD8αβ, 0.1-0.2 $x10^6$ cells were plated in 1 mL of R10 media with 1 mL of lentiviral supernatant (not concentrated), followed by spinfection at 800 x *g* for 1.5 h and RT with 0.5 mg/mL of LentiBOOST (Sirion Biotech, Cambridge, MA, USA). The cells were incubated at 37°C overnight and media replaced the following morning. Codon-optimized MC.7.G5 (18), MC.27.759S, K8T-1, K8T-2, MR1Ts JMA, DGB129, TC5A87 and ACA14 (17), and MAIT cell AF-7 (56) TCRs were expressed from the pSF plasmid, with the TRA and TRB chains separated by a self-cleaving T2A, and a P2A between the TRB gene and rCD2 co-marker: XbaI-Kozak-TRA-T2A-TRB-XhoI-P2A-rCD2-SalI-Stop. TCR transgene expression in TPR Jurkat cells was confirmed using antibodies for detection of surface expression of CD3 (clone BW264/56, PerCP conjugated, Miltenyi Biotec) and αβTCR (clone IP26, FITC conjugated, BioLegend), and additionally with TRBV specific antibodies for the MC.7.G5 TCR (Vbeta11/TRBV25, clone C21, APC conjugated, Beckman Coulter), MC.27.759S and K8T-1 TCRs (Vbeta8/TRBV12-4, clone 56C5.2, FITC conjugated, Beckman Coulter), and K8T-2 TCR (Vbeta3/TRBV28, clone CH92, FITC conjugated, Beckman Coulter). The rCD2 co-marker was used for magnetic based enrichments using APC (clone OX34, Abcam) or PE (clone OX34, BioLegend) conjugated rCD2 antibodies and anti-APC or anti-PE microbeads (Miltenyi Biotec) following the manufacturer's instructions.

Testing CD8αα and CD8αβ in Jurkat cells

To control for levels of TCR expression we took TPR Jurkat cells with no CD8 and already expressing MC.7.G5, MC.27.759S or AF-7 TCRs (with rCD2 co-marker), then introduced CD8α or CD8αβ. Human CD8α (NM_001768) and CD8β (NM_172213), with the latter separated by the P2A self-

cleaving sequence, were subcloned into the multiple cloning site of the Snap Fast plasmid (pSF). Sequence integrity was confirmed by Sanger Sequencing and expression in Jurkat cells confirmed by flow cytometry using CD8α (clone SK1, BV510 conjugate, BioLegend) and CD8β (clone QA20A40, APC conjugate, BioLegend) antibodies. CD8 magnetic microbeads were used to enrich $CD8^+$ cells according to the manufacturer's instructions (Miltenyi Biotec).

CD19 chimeric antigen receptor

Second generation CD19 chimeric antigen receptor (CAR) (57) with truncated Epidermal Growth Factor Receptor (tEGFR) co-marker was cloned into a pSF plasmid using Xba1 and Xho1: Xba1-Kozak-IgGK VIII signal peptide-Light chain-(G4S)3 linker-Heavy chain-IgG4 hinge-CD28 transmembrane-CD28-CD3ζ-P2A-Xho1-tEGFR-SalI-STOP. Jurkat cells were transduced as described above for TCR lentivirus and enriched using anti-tEGFR APC conjugated antibody (clone AY13, BioLegend) and anti-APC magnetic microbeads according to the manufacturer's instructions (Miltenyi Biotec).

TCR replacement

The MC.7.G5 TCR (codon optimized to avoid subsequent knockout) was expressed from the pSF plasmid, as detailed above for transduction of Jurkat cells. Oligonucleotides for the sgRNA sequence of *TRBC1 and TRBC2* genes (5'-CAAACACAGCGACCTCGGGTGGG) (23) were designed and cloned into the pLentiCRISPR v2 plasmid, according to guidelines associated with the pLentiCRISPR v2 plasmid available via the Addgene website. We also adapted the original pLentiCRISPR v2 by removing the puromycin resistance gene and replacing it with tEGFR to act as a co-marker. The replacement was achieved using the NEBuilder HiFi Assembly kit according to manufacturer's recommendations (New England Biolabs). Primers were designed with 40bp overlapping ends, and the reaction was carried out using 50 ng vector with 1:4 vector:insert ratio. Assembly reactions were incubated at 50°C for 1 h and integrity was confirmed with Sanger Sequencing. We used the puromycin plasmid for primary T-cells and the tEGFR plasmid for E6.1 Jurkat cells. For primary T-cells, CD8 T-cells were purified from healthy donors using anti-CD8 microbeads (Miltenyi Biotec) according to the manufacturer's guidelines, followed by overnight incubation with CD3/CD28 Dynabeads (Life Technologies) at 3:1 bead:T-cell ratio in T-cell media (R10 with 2 mM L-glutamine, 25 ng/ml IL-15 (Miltenyi Biotec), 200 IU/mL IL-2 (Proleukin®; Prometheus, San Diego, CA), 1X non-essential amino acids solution, 1 mM sodium pyruvate and 10 mM HEPES buffer (all from Merck, St Louis, Missouri, USA, unless stated otherwise). Per transduction, 1 $x10^6$ CD8 T-cells were used in 1 well of a 48 well plate, with concentrated lentivirus from one T175 flask. On the day of transduction, the media was removed from the T-cells, replaced with 0.5 mL of T-cell media and 0.2 mL of concentrated lentivirus added, then spinfected at 800 x *g* for 1.5 h and RT with 1 mg/mL of LentiBOOST (Sirion Biotech). For TCR replacement, concentrated

virus generated from one T175 flask of the TCR transgene, and one T175 flask of the *TRBC* knockout lentivirus, were added to the same well of T-cells. For comparison, wells of T-cells only receiving TCR transgene virus (no knockout) also received media to make the same volume as TCR replacement wells. The CD3/CD28 Dynabeads were removed on day 7 using a DynaMag-15 (Life Technologies). Between days 8 and 13 *TRBC* knockout T-cells were selected using 2 µg/mL of puromycin (Invitrogen). Transduced T-cells were enriched on day 10 with anti-rCD2 PE antibody (clone OX34, BioLegend) and anti-PE microbeads according to the manufacturer's instructions (Miltenyi Biotec). Between day 14 and 18 the selected and enriched T-cells were expanded, using 0.2 $\times 10^6$ T-cells per well of a 24 well plate and 4 $\times 10^6$ irradiated (3100 Gy) PBMCs from three allogenic donors, provided by the Welsh Blood Service, and 1 µg/mL of L-phytohemagglutinin (PHA) (Remel, ThermoFisher Scientific) in 2 mL T-cell media with 20 IU/mL of IL-2 and 25 ng/mL of IL-15. T-cells were fed thrice weekly by changing 50% of the media, and rapidly dividing cells split 1-3 times over multiple 24 wells. T-cells were used for functional assays from day 14 onwards post expansion.

E6.1 Jurkat cells (cultured in R10 as suspension cells) were co-transduced with *TRBC1/2* knockout tEGFR lentivirus and enriched using anti-tEGFR APC conjugated antibody (clone AY13, BioLegend) and anti-APC magnetic microbeads (Miltenyi Biotec). Knockout of the tEGFR+ Jurakt cells was confirmed with CD3 (clone BW264/56, PerCP conjugated, Miltenyi Biotec) and $\alpha\beta$TCR (clone IP26, FITC conjugated, BioLegend) antibodies. MC.7.G5, MC.27.759S, K8T-1 and K8T-2 TCRs with rCD2 co-marker was expressed then enriched using anti-rCD2 PE conjugated antibody (clone OX34, BioLegend) and anti-PE magnetic microbeads (Miltenyi Biotec). MC.7.G5 expression was tested using anti-rCD2 PE conjugated antibody (clone OX34, BioLegend) and a TRBV specific antibody (Vbeta11/TRBV25, clone C21, APC conjugated, Beckman Coulter). K8T-2 expression was tested using anti-rCD2 PE conjugated antibody (clone OX34, BioLegend) and a TRBV specific antibody (Vbeta3/TRBV28, clone CH92, FITC conjugated, Beckman Coulter). E6.1 TCR staining and CD69 assay gating strategies in **Supplementary Figure 6B**.

CD69 assays

For CD69 assays, 5 $\times 10^4$ Jurkat cells and 1 $\times 10^5$ target cells were plated in 96 U-well plates in 200 µL of R10, then incubated for 4h or 24h. For testing MR1 ligands, Acetyl-6-formylpterin (Ac-6-FP) (Schricks Laboratories, Switzerland) and 5-amino-6-D-ribitylaminouracil (5-A-RU) (TargetMol Chemicals Inc., Boston, MA, USA) were added directly to the assays well to give 10 µg/mL. CD3/CD28 Dynabeads (2 µL per well) (ThermoFisher Scientific) or 0.02 µg/mL Phorbol 12-Myristate 13-Acetate (PMA) (Promega, Wisconsin, USA) were used as positive controls for TCR-transduced and untransduced Jurkat cells respectively. Cells were stained with viability stain ViViD (ThermoFisher Scientific), then the following antibodies: CD8 APC Vio770 (to identify the TPR Jurkat cells) (clone BW135/80, Miltenyi Biotec), rCD2 PE (TCR co-marker) (clone OX34, BioLegend), or

tEGFR APC (CD19-CAR or *TRBC* knockout co-marker) (clone AY13, BioLegend), and CD69 APC (clone FN50, BioLegend) or CD69 PE (clone TP1.55.3, Beckman Coulter). Human FcR block was used according to the manufacturer's instruction (Miltenyi Biotec) when using monocytes, B-cells or leukemia cell lines as the target cells. Gating for TPR Jurkat cells +/- transduced TCR: (FSC-H versus SSC-H), single cells (FSC-A versus FSC-H), viable cells (SSC-H versus ViViD stain low/negative), rCD2 versus CD8, then CD69 APC for the mean fluorescence intensity values (**Supplementary Figure 6C**). Gating for TPR Jurkat cells +/- CD19-CAR: (FSC-H versus SSC-H), single cells (FSC-A versus FSC-H), viable cells (SSC-H versus ViViD stain low/negative), tEGFR versus CD8, then CD69 PE for the mean fluorescence intensity values (**Supplementary Figure 6C**). Gating for E6.1 Jurkat cells with MC.7.G5, MC.27.759S, K8T-1 or K8T-2 TCRs +/- *TRBC* knockout: (FSC-H versus SSC-H), single cells (FSC-A versus FSC-H), viable cells (SSC-H versus ViViD stain low/negative), rCD2 versus tEGFR, then CD69 PE for the mean fluorescence intensity values (**Supplementary Figure 6B**). Jurkat cells alone and positive control values shown is **Supplementary Excel file**. Acquisition was performed on a ACEA NovoCyte 3005 with NovoSampler pro (ACEA, Agilent, Santa Clara, CA, USA).

Flow cytometry-based cytotoxicity assay

Flow-based killing assays were performed as previously described (18). Briefly, target cells were co-incubated with and without T-cells for 24 h in 96U-well plates containing T-cell media with 20IU/mL IL-2 and 25 ng/mL IL-15. On the day of harvest, 1-3 x10^4 C1R GFP$^+$ cells added to each assay well, followed by incubation with FcR blocking reagent (Miltenyi Biotec) and staining with viability stain ViViD (ThermoFisher Scientific), CD3 BV711 conjugated (clone UCHT1, BioLegend), CD8 APC conjugated (clone BW135/80, Miltenyi Biotec), CD4 APC conjugated (clone VIT4, Miltenyi Biotec) and TRBV25 APC conjugated (clone C21, Beckman Coulter) antibodies. For identification of healthy PBMC cell subsets further antibodies were included: CD14 APC Cy7 conjugated (clone HCD14, BioLegend) and CD19 PE conjugated (HIB19, BioLegend). Acquisition was performed on a ACEA NovoCyte 3005 with NovoSampler pro (ACEA, Agilent, Santa Clara, CA, USA). T-cells (CD3, CD8, CD4 and TRBV25) and dead/dying cells were excluded from analysis, leaving viable target cells and GFP$^+$ C1Rs to calculate the percentage of killing using the following equation. Gating strategies are shown in **Supplementary Figure 7A&B**.

$$killing\ = 100\ - \left(\left(\frac{with\ T\ cells\text{: } \%\ or\ cell\ events\ of\ target\ cells \div \%\ or\ cell\ events\ of\ C1R\ GFP}{without\ T\ cells\text{: } \%\ or\ cell\ events\ of\ target\ cells \div \%\ or\ cell\ events\ of\ C1R\ GFP} \right) \times 100 \right)$$

T107 assay

T-cells were rested in R5 (as for R10 with 5% FBS) for 24 h before setting up the assay to help reduce spontaneous activation. T-cells (3 x10^4) and target (6 x10^4) cells were co-incubated for 4-6 h with 30 µM TNF-processing inhibitor (TAPI)-0 (Merck), and antibodies directed against TNF PE Vio770

(clone cA2, Miltenyi Biotec) and CD107a PE (clone H4A3, BD Biosciences). Following co-incubation, cells were washed with PBS then stained with viability stain ViViD for 5 min at RT, FcR blocking reagent (Miltenyi Biotec) used according to the manufacturer's instructions (Miltenyi Biotec), and without washing antibodies against CD3 (clone BW264/56, PerCP conjugated ,Miltenyi Biotec), CD8 (clone REA734, APC conjugated, Miltenyi Biotec) and CD4 (clone VIT4, FITC conjugated, Miltenyi Biotec) were added and incubated for 20 min on ice. For CD8 and CD161 phenotyping, CD8α (clone SK1, BV510 conjugate, BioLegend), CD8β (clone QA20A40, APC conjugate, BioLegend), and CD161 (clone 191B8, PE Vio770 conjugate, Miltenyi Biotic) antibodies were used. Cells were washed in PBS and acquired immediately on a flow cytometer or fixed with 2% formaldehyde for acquisition within 24 h. Acquisition was performed on a ACEA NovoCyte 3005 with NovoSampler pro (ACEA, Agilent, Santa Clara, CA, USA). Gating was for lymphocytes (FSC-A/H versus SSC-A/H), single cells (FSC-A versus FSC-H), viable $CD3^+$ cells (VIVID low/negative), $CD3^+/CD8^+$ or $CD3^+/CD4^+$, then displayed as TNF versus CD107a. For CD8 and CD161 phenptyping: lymphocytes (FSC-A/H versus SSC-A/H), single cells (FSC-A versus FSC-H), viable $CD3^+$ cells (VIVID low/negative), TNF versus CD107a, then CD8α versus CD8β, or CD161. Gating strategies in **Supplementary Figure 7C**.

Enzyme Linked Immunosorbant assay (ELISA)

T-cells were 'rested' overnight in R5 medium (R10 but with 5% FBS serum) to help reduce spontaneous release of chemokines and cytokines. 3 $x10^4$ T-cells were used in 96U well plates at 1:2 ratio with antigen presenting cells or target cells in R5 medium and incubated overnight. Supernatants were harvested and high-binding half-area ELISA microplates (Greiner, Frickenhausen, Germany) used in conjunction with TNF or Granzyme B DuoSet ELISA kits according to the manufacturer's instructions (R&D Systems, Abingdon, UK). T-cells and targets cells were incubated alone to check for spontaneous release of TNF or Granzyme B. PHA (10 µg/mL) (Remel, ThermoFisher Scientific) or CD3/CD28 Dynabeads (Life Technologies) were used as a positive control for the T-cells (alone and positive control values shown is **Supplementary Excel file**).

Data display, analysis and statistics

Unless stated otherwise, all data were displayed using GraphPad Prism software. Statistical tests were performed in R and included multivariate permutation test for paired comparison (https://rdrr.io/cran/CNPS/), Shapiro-Wilk normality and paired T-test, and Wilcoxon signed-rank test (not normally distributed). TCR V-J usage plots were generated using VDJ tools (58). Error bars depicting SEM are displayed when triplicate conditions were performed. Flow cytometry data analyzed with FlowJo software (Tree Star Inc., Ashland, Oregon, US) or NovoExpress (Agilent, Santa Clara, CA, USA).

Supplementary References

The references above conform with the manuscript reference numbers but include the additional eleven references listed below:

48. Andersen R, Donia M, Ellebaek E, Borch TH, Kongsted P, Iversen TZ, et al. Long-Lasting Complete Responses in Patients with Metastatic Melanoma after Adoptive Cell Therapy with Tumor-Infiltrating Lymphocytes and an Attenuated IL2 Regimen. *Clin Cancer Res.* 2016;22(15):3734-45.
49. Muller TR, Schuler C, Hammel M, Kohler A, Jutz S, Leitner J, et al. A T-cell reporter platform for high-throughput and reliable investigation of TCR function and biology. *Clin Transl Immunology.* 2020;9(11):e1216.
50. Laugel B, Lloyd A, Meermeier EW, Crowther MD, Connor TR, Dolton G, et al. Engineering of Isogenic Cells Deficient for MR1 with a CRISPR/Cas9 Lentiviral System: Tools To Study Microbial Antigen Processing and Presentation to Human MR1-Restricted T Cells. *J Immunol.* 2016;197(3):971-82.
51. Galloway SAE, Dolton G, Attaf M, Wall A, Fuller A, Rius C, et al. Peptide Super-Agonist Enhances T-Cell Responses to Melanoma. *Frontiers in immunology.* 2019;10:319.
52. Bolotin DA, Poslavsky S, Mitrophanov I, Shugay M, Mamedov IZ, Putintseva EV, et al. MiXCR: software for comprehensive adaptive immunity profiling. *Nature methods.* 2015;12(5):380-1.
53. Shugay M, Bagaev DV, Zvyagin IV, Vroomans RM, Crawford JC, Dolton G, et al. VDJdb: a curated database of T-cell receptor sequences with known antigen specificity. *Nucleic Acids Res.* 2018;46(D1):D419-D27.
54. Tanno H, McDaniel JR, Stevens CA, Voss WN, Li J, Durrett R, et al. A facile technology for the high-throughput sequencing of the paired VH:VL and TCRbeta:TCRalpha repertoires. *Sci Adv.* 2020;6(17):eaay9093.
55. Sanjana NE, Shalem O, and Zhang F. Improved vectors and genome-wide libraries for CRISPR screening. *Nature methods.* 2014;11(8):783-4.
56. Tilloy F, Treiner E, Park SH, Garcia C, Lemonnier F, de la Salle H, et al. An invariant T cell receptor alpha chain defines a novel TAP-independent major histocompatibility complex class Ib-restricted alpha/beta T cell subpopulation in mammals. *J Exp Med.* 1999;189(12):1907-21.
57. Nicholson IC, Lenton KA, Little DJ, Decorso T, Lee FT, Scott AM, et al. Construction and characterisation of a functional CD19 specific single chain Fv fragment for immunotherapy of B lineage leukaemia and lymphoma. *Mol Immunol.* 1997;34(16-17):1157-65.
58. Murshudov GN, Skubak P, Lebedev AA, Pannu NS, Steiner RA, Nicholls RA, et al. REFMAC5 for the refinement of macromolecular crystal structures. *Acta Crystallogr D Biol Crystallogr.* 2011;67(Pt 4):355-67.
